# The impact of targeted local outreach clinics to improve COVID-19 vaccine uptake: controlled interrupted time series in South West England

**DOI:** 10.1186/s13690-024-01341-1

**Published:** 2024-08-07

**Authors:** Tim Jones, Huzaifa Adamali, Maria Theresa Redaniel, Frank de Vocht, Kate Tilling, Charlie Kenward, Yoav Ben-Shlomo, Sam Creavin

**Affiliations:** 1 The National Institute for Health Research Applied Research Collaboration West (NIHR ARC West), University Hospitals Bristol NHS Foundation Trust, Bristol, BS1 2NT UK; 2 Population Health Sciences, Bristol Medical School, University of Bristol, Bristol, BS8 2PS UK; 3 NHS Bristol, North Somerset, and South Gloucestershire Integrated Care Board, Bristol, UK; 4 North Bristol NHS Trust, Southmead Hospital, Bristol, UK

**Keywords:** Vaccines, Healthcare disparities, Interrupted Time Series Analysis, SARS-CoV-2

## Abstract

**Background:**

Outreach clinics were part of efforts to maximise uptake in COVID-19 vaccination.

**Methods:**

We used controlled interrupted time series, matching on age, sex, deprivation and vaccination eligibility date, to determine the effect of outreach clinics on time to first COVID-19 vaccine, using a population-based electronic health record database of 914,478 people, from December 2020 to December 2021; people living within 1 mile of each outreach clinics were exposed.

**Results:**

50% of 288,473 exposed citizens were white British, and 71% were aged 0–49 years. There was no evidence for an overall statistically significant increase in cumulative percentage vaccinated due to the outreach clinic at 6 weeks, with an overall pooled effect estimate of -0.07% (95% CI: -1.15%, 1.02%). The pooled estimate for increased cumulative vaccine uptake varied slightly depending on how the analysis was stratified; by ethnic group it was − 0.12% (95% CI: -0.90%, 0.66%); by age group it was − 0.06% (95% CI: -0.41%, 0.28%); and by deprivation it was 0.03% (95% CI: -0.74%, 0.79%).

**Conclusions:**

Living within a mile of an outreach clinic was not associated with higher vaccine uptake. Evaluation of future outreach clinics should consider the relative importance of travel amongst other barriers to accessing vaccines.

**Supplementary Information:**

The online version contains supplementary material available at 10.1186/s13690-024-01341-1.

**Table:** 

**Text box 1. Contributions to the literature**
• Improving vaccine uptake is an important focus for efforts of many health systems to reduce health inequalities.
• Outreach vaccine clinics are one intervention that have been proposed to help improve vaccine uptake.
• We analysed the effect of outreach vaccine clinics, which were set-up during the COVID pandemic, on COVID vaccine uptake.
• We did not find any effect of outreach clinics on vaccine uptake and health commissioners should not assume that outreach clinics alone will improve vaccine uptake.

## Background

The COVID-19 pandemic has revealed and worsened health inequalities [1, 2]. COVID-19 mortality rates have been highest in areas of socioeconomic disadvantage, and ethnic minorities (though people in ethnic minorities often are people of the global majority) [3] and there is also evidence of a widening in long term health inequalities [4].

COVID-19 was first reported in the UK in January 2020 [5], and a vaccine was available by December 2020 [6]. The Joint Committee on Vaccination and Immunisation (JCVI) prioritised groups according to the ‘green book’ criteria [7]. The criteria comprise nine priority groups [7], (1 being the highest priority) Residents living in, and staff working in, care homes for older adults, [2] 80 + years of age; frontline health and social care workers [3] 75 + years of age [4] 70 + years of age; individuals aged 16 to 69 years in a high risk group [5] 65 + years of age [6] Adults aged 16 to 65 years in an at-risk group (7–9 sequentially) aged 60 + years, 55 + years, and 50 + years. By 18 June 2021 all adults over 18 years were eligible for a first vaccination [8]. A COVID-19 vaccine passport for some public venues was mandatory between 19 July 2021 and 27 January 2022 [9].

There is inequality in COVID-19 vaccine uptake across ethnic minority groups [10] and relatively high proportions of people reporting vaccine hesitancy even amongst healthcare workers [11]. Reasons for vaccine hesitancy include lack of confidence in the vaccine, complacency about the risks, unclear and negative messages confusing public health measures, and inconvenient access to a vaccine [12, 13]. Vaccine hesitancy may be addressed by community engagement and tailored communication [14], monetary incentives, and technology-based health literacy programmes [15]. Understanding the real-world impact of community interventions, such as local outreach clinics, on vaccine uptake, will inform local and national future population health initiatives.

The Bristol, North Somerset and South Gloucestershire (BNSSG) clinical commissioning group (CCG; now integrated care boards [ICBs]) COVID-19 Maximising Uptake Programme coordinated efforts to increase COVID-19 vaccine uptake among those at risk of low uptake, including people of ethnic minority and non-English speakers [16]. Outreach clinics developed partnerships with vulnerable communities, and included vaccine myth-busting webinars, development of vaccine information resources in different languages, community led clinics and health coaching, as well as providing vaccinations.

The aim of this study is to understand the extent to which local outreach clinics in BNSSG were effective in improving vaccine uptake in the clinic environs [17, 18]. Our hypothesis was that outreach clinics would help to improve vaccine uptake.

## Methods

This is a longitudinal observational study using prospectively-collected routine administrative information about patients registered with UK GP practices in BNSSG. Almost all patients in the UK are registered at a GP practice [19]. It is reported according to the RECORD [20] extension to STROBE guidelines.

### Data sources

We used an extract from the BNSSG system-wide dataset [21], which brings together information from primary, secondary (hospitals), community (e.g., community nursing), and mental health care within BNSSG. The extract included information on sex, age, ethnicity, primary language, comorbidities, and COVID-19 vaccination dates (but not vaccination location) for people registered with contributing local GP (General Practitioner or family doctor) practices between December 2020 and December 2021 (*n* = 914,478). The available data included all patients registered at a GP in this health system, and represents over 90% of the BNSSG population of roughly 1 million people. Our data comprised monthly snapshots linked on patient ID; people could enter or leave the dataset in any month as citizens respectively moved into or out of the health system.

### Data linkage

South-West Clinical Support Unit (a non-governmental public body) performed the record linkage within the system-wide dataset, matching unique records on NHS number. The system-wide dataset was linked to rural/urban classification [22] and deprivation [23] information by the study team, using small geographic areas with a mean population of 1,500 (lower super output area of residence, LSOA [24]) for each person. We included all NHS users in our geographic region.

### COVID-19 vaccine eligibility

We categorised people into JCVI priority groups according to the ‘green book’ criteria, including age [7], as outlined in UK timelines for vaccination. We did not have data on occupation, which led to some misclassification of eligibility dates. This is because health workers were given high prioritisation for vaccination irrespective of age, and therefore should have been vaccinated earlier than suggested by their age. Without information on occupation, we may have assumed they were eligible later than they actually were. Based on 2021 UK census data, around 10% of the population in Bristol work in human health activities [25].

### Local COVID-19 vaccine interventions

We were given a list of 50 local COVID-19 vaccine outreach clinics by BNSSG CCG, occurring on 196 dates, including location (postcode), date, and target population (e.g., homeless, ethnic minority groups). We excluded outreach clinics where we could not identify target citizens (homeless, refugees, asylum seekers, people with drug and alcohol addiction, learning disabilities, or serious mental illness). Additionally, we excluded the outreach targeting young people at a large shopping centre (Cabot Circus), because it was difficult to identify ‘exposed’ individuals as people travel from surrounding areas to go shopping. The remaining 18 intervention locations with 53 intervention dates all focussed on people of ethnic minority or whose primary language was not English, being located in areas with a higher proportion of these populations.

### Distances between local COVID-19 vaccine outreach clinics and people’s homes

We obtained Ordinance Survey map coordinates for each outreach clinic from the national statistics postcode lookup (NSPL) [26], and for each person’s home using population-weighted centroids for each citizen’s LSOA from the Office for National Statistics [27]. We calculated distances in straight lines.

### Ethnicity

Ethnicity was self-reported and provided using 222 different text labels. These were categorised into ethnic groups as described in Supplementary Table T1 [28].

### Exposure and matched non-exposed

For each of the 18 intervention sites, we considered citizens as exposed to an outreach clinic if they lived within 1 mile of the outreach site at the time of the clinic since we judged this distance is generally possible to walk in 15–20 min. For each included outreach clinic, we separately matched a control group specific to that clinic, chosen from the pool of people registered with GP practices within BNSSG but living further than a mile (no upper limit) from any outreach clinic. We used 1:1 matching with replacement on age (+/- 2 years), and exact matching on sex, ethnicity, deprivation quintile, and date of eligibility for vaccine based on JCVI criteria. When there was more than one match for a control, we chose one randomly. We excluded people from the intervention group who had no matched control; on average this was 3.6% of each intervention cohort (SD: 0.5%). The resulting control group for each intervention site included the same number of people as that intervention group.

### Statistical analyses

We used letter codes to pseudonymise outreach clinics; the purpose of the study was not to report results for specific named sites, but to explore the impact of the intervention across all targeted clinic sites. We used COVID-19 vaccine first dose dates to calculate cumulative percentage of people exposed to each intervention having a first COVID-19 vaccination dose between 8th December 2020 (start of vaccine eligibility) and 31st December 2021, using Kaplan Meier [29].

For most outreach clinics, we used the first local outreach clinic date at that site as their date of intervention (index date). However, for some locations, the earliest clinic date fell during a levelling off in vaccination rates across BNSSG (April to June 2021) when it would be difficult to detect any impact of the intervention. For outreach clinics like this, when they had multiple clinic dates (areas D, E, and F), we selected the last clinic date as their index date.

We conducted controlled interrupted time series (CITS) analysis [30, 31] to explore the change in cumulative vaccine uptake at 6 weeks after outreach clinic (compared to expectation from the pre-clinic trend) for exposed and control groups. We used six weeks after for two reasons: there was a hope that vaccine clinic interventions would have a ripple effect beyond the clinic itself; and in wave one of the vaccine campaign there were occasionally delays in updating vaccination records in the health care record due to the ongoing pandemic, and on occasions up to six weeks may have passed before someone’s health record was updated, though the date of vaccination we were given would have been recorded accurately. The difference-in-differences between exposed and control groups was considered to be the impact of the outreach clinic. We did this separately for each of the 18 intervention sites. Detailed CITS methods are included in the supplementary materials.

An assumption of CITS is that the trend lines are parallel before the interruption. We tested this assumption by including an interaction between the pre-clinic slopes of the outreach area and control area.

### Sensitivity and validation analyses

In sensitivity analyses, we further restricted local outreach clinics to a sub-set we deemed most likely to focus on ethnic groups either by religious affiliation or country of origin. We stratified analyses by: ethnicity, comparing white British people to ethnic minority groups; age group; and deprivation quintile.

## Results

### Descriptive information

**Fig. 1:**
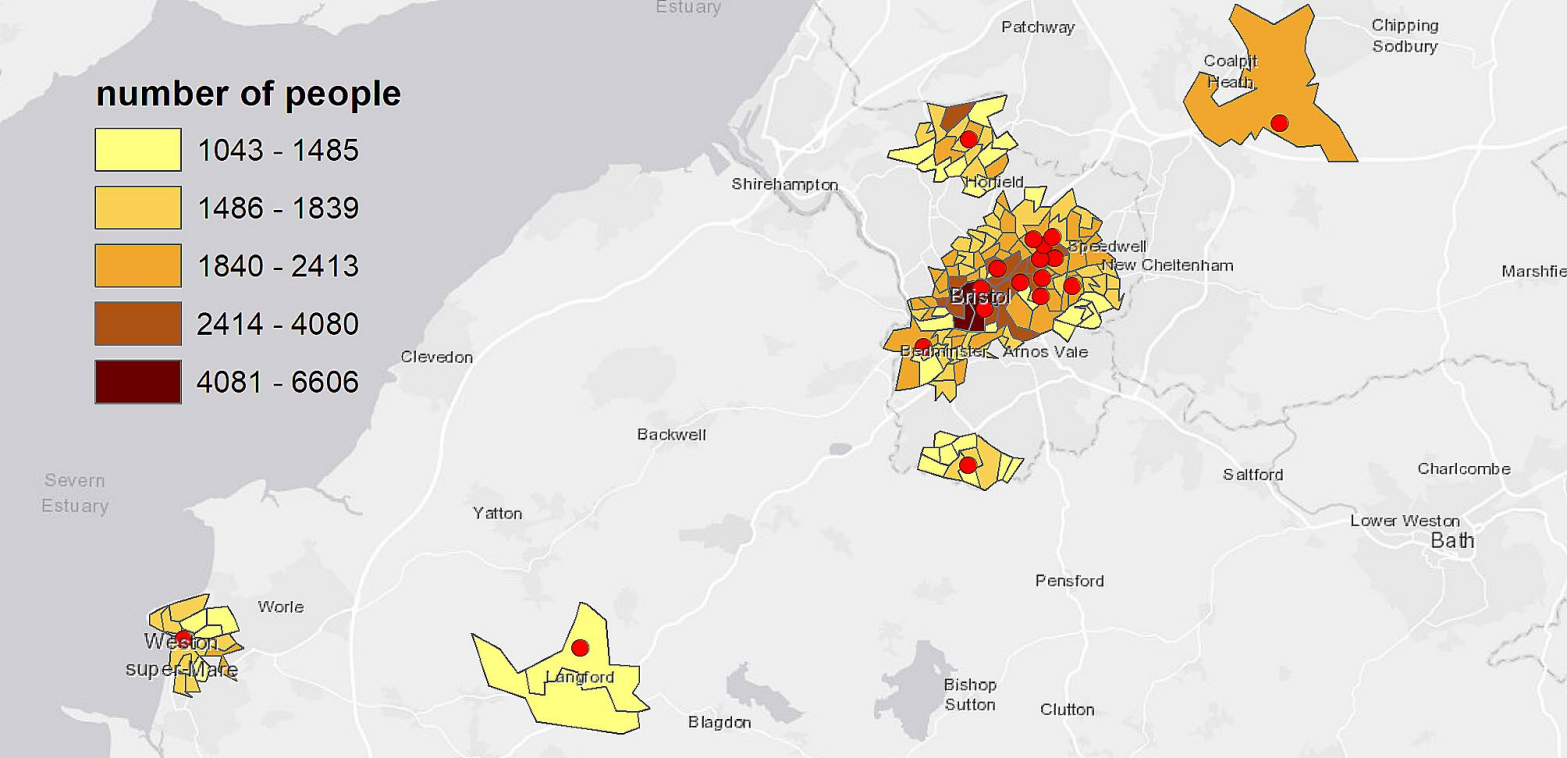
Map showing the location of local COVID-19 vaccine outreach clinics targeting people of ethnic minority and people without English as primary language, and counts of people living within 1 mile of these outreach clinics

Figure 1 shows the COVID-19 outreach clinic locations on a map, with colours representing counts of people living in each LSOA. Supplementary Table T2 provides summary information about the outreach clinics (number of clinics at location, earliest and latest dates of intervention), and Supplementary Table T3 shows which outreach clinics are within a mile from each other. There was considerable overlap, with only 6 outreach clinics being more than a mile from any others (see also Fig. 1).

Table 1 summarises characteristics of people living within 1 mile of an outreach clinic and people living further than 1 mile from any outreach clinic (pool of potential controls). People living within 1 mile of an outreach clinic were, on average, younger, more likely to belong to an ethnic group, more deprived, and less likely to have English as a primary language than those living further out, as one might expect given that these were selected populations for targeting.

**Table 1:** Characteristics of people in our systemwide dataset cohort living within 1 mile of an included intervention or living more than 1 mile from any intervention

	People > 1 mile away from any intervention		People within 1 mile of an intervention	
	n	%	n	%
Population	626,005	100.00	288,473	100.00
Sex				
*men*	316,538	50.56	142,393	49.36
*women*	309,446	49.43	146,060	50.63
*missing*	21	0.00	20	0.01
Age group				
*0–49*	346,749	55.39	205,437	71.22
*50–54*	49,165	7.85	17,647	6.12
*55–59*	48,538	7.75	16,498	5.72
*60–64*	40,872	6.53	12,917	4.48
*65–69*	35,133	5.61	10,036	3.48
*70–74*	35,791	5.72	9,145	3.17
*75–79*	28,919	4.62	6,651	2.31
*80+*	40,838	6.52	10,142	3.52
Ethnic Group				
*white british*	408,336	65.23	144,409	50.06
*white irish*	2,902	0.46	2,323	0.81
*other white*	42,416	6.78	33,817	11.72
*indian*	6,749	1.08	4,783	1.66
*pakistani*	2,939	0.47	3,858	1.34
*bangladeshi*	1,320	0.21	1,172	0.41
*chinese*	4,539	0.73	4,765	1.65
*other asian*	5,584	0.89	4,847	1.68
*black caribbean*	2,019	0.32	3,502	1.21
*black african*	5,273	0.84	10,361	3.59
*other black*	1,323	0.21	2,035	0.71
*mixed*	8,025	1.28	6,934	2.40
*gypsy/roma/traveller*	135	0.02	153	0.05
*other*	2,902	0.46	5,889	2.04
*missing*	123,789	19.77	59,625	20.67
Primary language				
*english*	165,262	26.40	98,243	34.06
*non-english*	21,495	3.43	24,639	8.54
*missing*	439,248	70.17	165,591	57.40
Charlson Comorbidities				
*0*	490,741	78.39	241,330	83.66
*1*	88,271	14.10	31,631	10.96
*2–3*	39,695	6.34	12,897	4.47
*4+*	7,298	1.17	2,615	0.91
Rural / Urban				
*rural*	62,429	9.97	4,372	1.52
*urban*	562,914	89.92	284,101	98.48
*missing*	662	0.11	0	0.00
Deprivation				
*1 – most deprived*	69,181	11.05	90,742	31.46
*2*	86,124	13.76	79,017	27.39
*3*	104,059	16.62	50,959	17.67
*4*	157,577	25.17	47,972	16.63
*5 – least deprived*	208,374	33.29	19,783	6.86
*missing*	690	0.11	0	0.00
JCVI Group				
*1*	2,802	0.45	1,500	0.52
*2*	38,607	6.17	9,093	3.15
*3*	28,613	4.57	6,464	2.24
*4*	55,396	8.85	18,726	6.49
*5*	32,029	5.12	8,894	3.08
*6*	88,288	14.10	34,766	12.05
*7*	23,819	3.80	7,388	2.56
*8*	31,632	5.05	10,511	3.64
*9*	34,913	5.58	12,342	4.28
*other*	289,906	46.31	178,789	61.98

Note: ‘People living within 1 mile of an intervention’ are those living within 1 mile of any of the 18 interventions included in the study. ‘People living > 1 mile away from any intervention’ is the entire pool of ‘potential’ controls. For each of the 18 interventions we then do matching using this pool of potential controls

### Impact of local COVID-19 vaccine outreach clinics on vaccine uptake

**Fig. 2:**
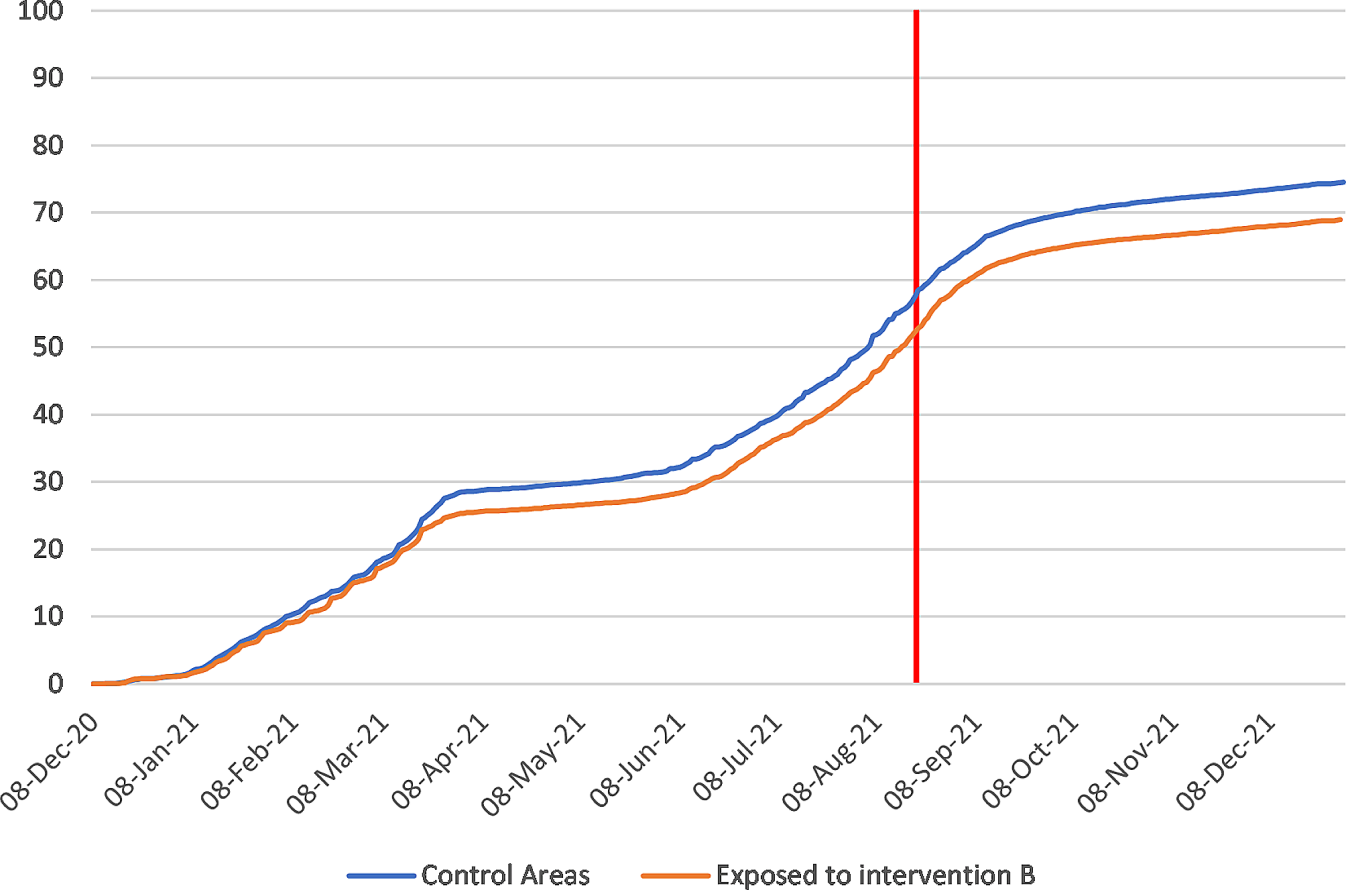
Cumulative COVID-19 vaccine uptake amongst people within 1 mile of the area B intervention and matched controls. Note: vertical red line is the intervention date

**Fig. 3:**
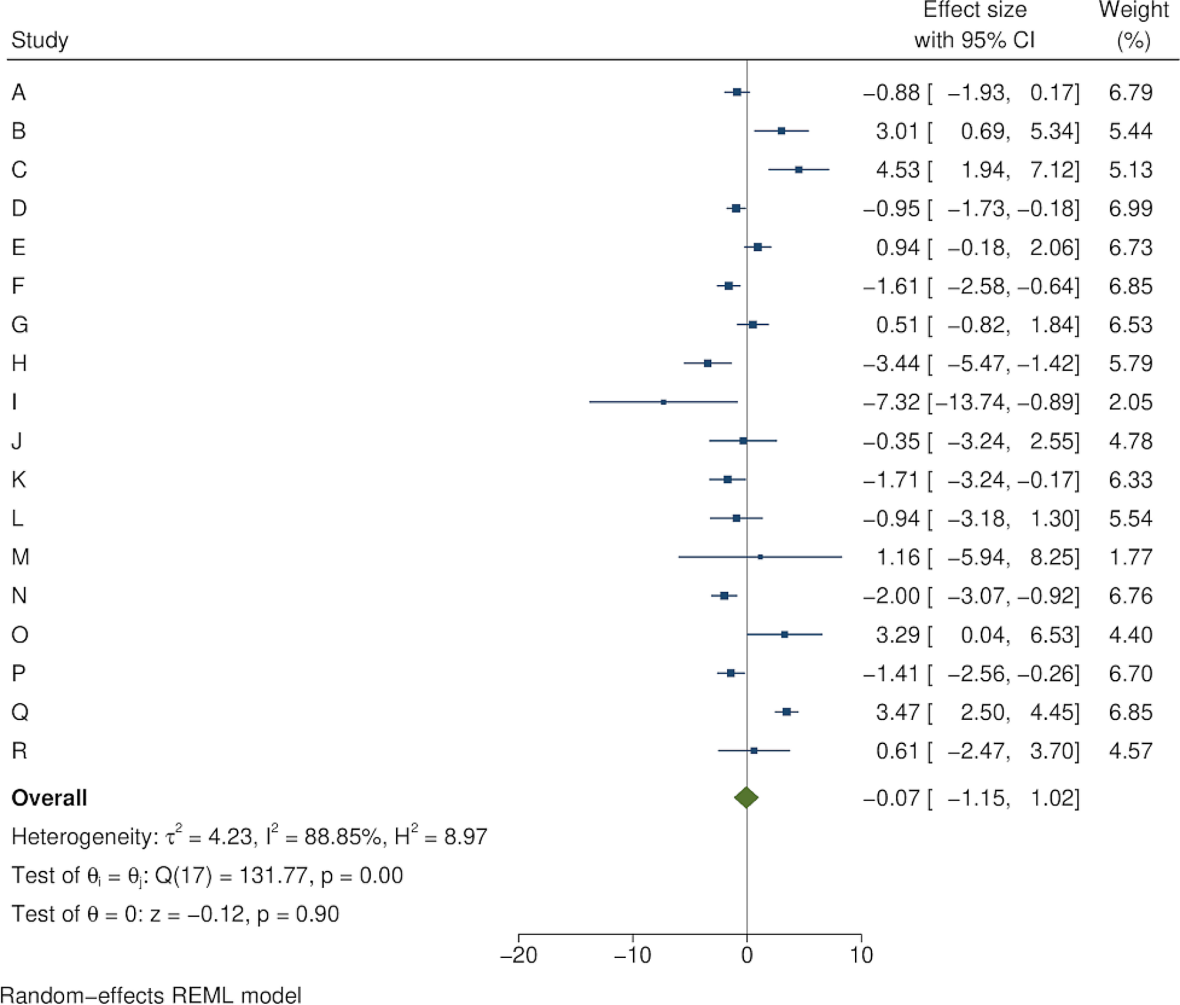
The impact of local COVID-19 vaccine outreach clinics on cumulative vaccination (%) at 6 weeks post-intervention compared to matched controls, overall

**Fig. 4:**
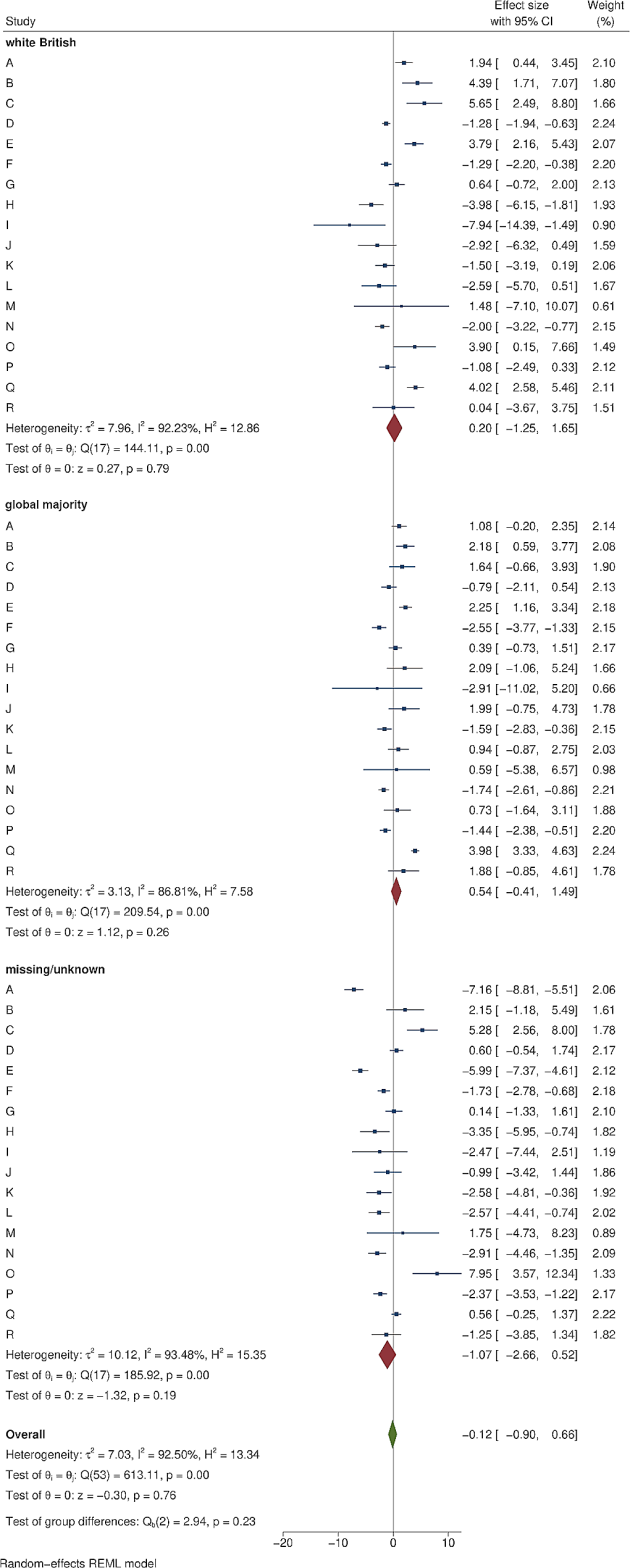
The impact of local COVID-19 vaccine outreach clinics on cumulative vaccination at 6 weeks post-intervention compared to matched controls, by ethnic group

**Fig. 5:**
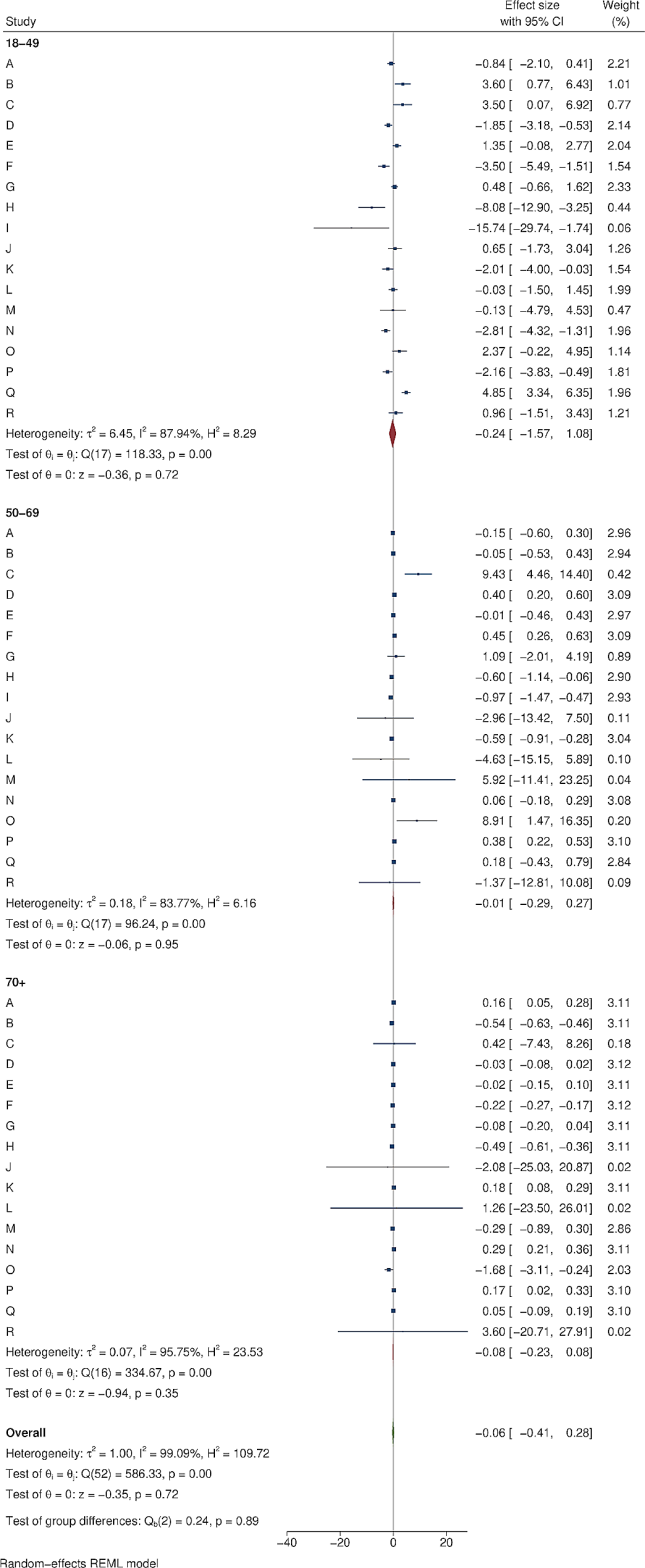
The impact of local COVID-19 vaccine outreach clinics on cumulative vaccination (%) at 6 weeks post-intervention compared to matched controls, by age group

**Fig. 6:**
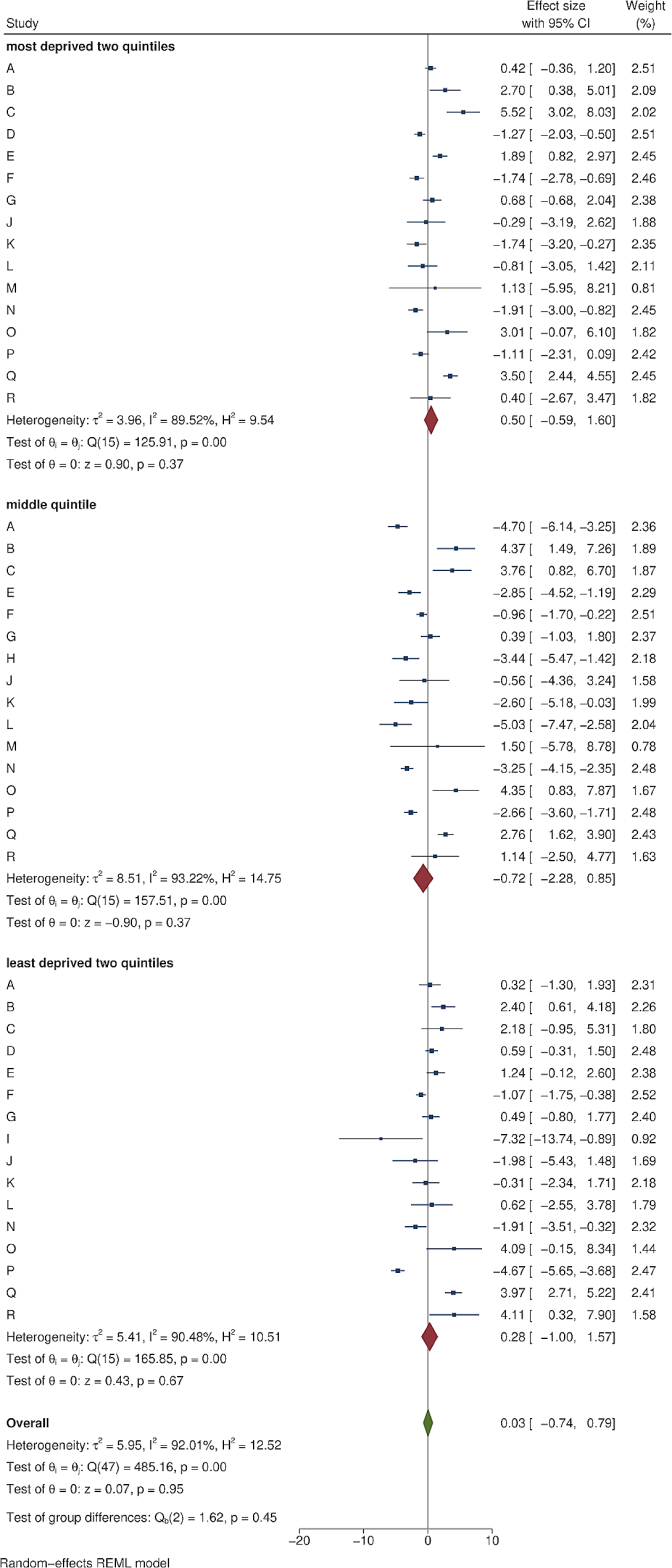
The impact of local COVID-19 vaccine outreach clinics on cumulative vaccination (%) at 6 weeks post-intervention compared to matched controls, by deprivation group

Figure 2 shows an example of cumulative COVID-19 vaccine uptake amongst people within 1 mile of the area B intervention and matched controls. There was initial uptake between December 2020 and March 2021, then a plateau before a further increase in uptake from June 2021 to September 2021. Information about each of the 18 local outreach clinics can be found in Supplementary Tables T2 and T3. The forest plots in Figs. 3, 4, 5 and 6 summarise the overall effect of local vaccine outreach clinics, as well as sub-group effects by ethnic group, age group, and deprivation. There was no evidence for an overall statistically significant increase in cumulative percentage vaccinated due to the outreach clinic at 6 weeks, with an overall pooled effect estimate of -0.07% (95% CI: -1.15%, 1.02%). The pooled estimate for increased cumulative vaccine uptake varied slightly depending on how the analysis was stratified; by ethnic group it was − 0.12% (95% CI: -0.90%, 0.66%); by age group it was − 0.06% (95% CI: -0.41%, 0.28%); and by deprivation it was 0.03% (95% CI: -0.74%, 0.79%). There was weak evidence, consistent with chance, of a deprivation gradient with stronger impact in the most deprived group (Fig. 6). There was large heterogeneity in results between outreach clinics (I^2^ > 80%).

## Discussion

### Main findings of this study

There was little evidence that the community-focussed COVID-19 outreach clinics improved cumulative vaccine uptake beyond vaccines delivered directly in the clinics, in the community residing within 1 mile of these clinics compared to matched groups further away from the outreach clinics. This was the case overall and within sub-groups by ethnicity, age, or deprivation. This could be because there was no measurable ‘ripple effect’ of the local clinics, that it is insensitive to our one-mile threshold, or that any affect was masked by the large number of varied opportunities for COVID-19 vaccination (e.g., in GP practices, and at mass vaccination sites in shopping centres and football stadiums) offered during the same period. Future vaccine campaign waves or mass vaccination programmes are unlikely to have so many different sites available for people to choose for vaccination, and once eligible people may select based on factors such as trust and ease of access. Commissioners of vaccine services should not rely on a vaccine uptake bonus from a ripple-effect from community vaccine clinics, and therefore need to factor in direct costs, proportional to the number of expected vaccines required to be delivered by community clinics to reach vulnerable populations.

### What is already known on this topic

Other studies have reported high vaccination uptake in people aged over 70 years living in England, with around 93% of these people having had one COVID-19 vaccine by 15 March 2021, but with lower uptake in black African (67%) and black Caribbean (74%) ethnic backgrounds; in that study [32] higher odds of not being vaccinated were associated with older age, greater deprivation, rented accommodation, being disabled, and living alone or in a multigenerational household [32]. Although we did not show a difference in rate of uptake for people of ethnic minority background based on their proximity to a vaccination site, we did find that vaccination uptake in this group continued to increase over time, whereas other studies found that differences in vaccination uptake widened over time between people of white British and Indian compared to black African and black Caribbean backgrounds [33]. Others have found that language often restricts accessibility and family members are required to help interpret [34].

A previous study in BNSSG [16] found increased vaccine uptake amongst ethnic minority groups compared to the population average, whereas we found little evidence for increased vaccine uptake for people within 1 mile of a clinic compared to those further away when matched on age, sex, and ethnicity. These findings could be consistent if outreach efforts improved vaccine uptake amongst hesitant groups more widely across the CCG area.

Others have reported that the most common vaccine hesitation factors were related to vaccine development, mistrust towards government, and misconceptions about safety [35, 36]. Tailored vaccine messaging, community outreach [14], and behavioural nudges [37], as well as monetary incentives [38] have been shown to improve vaccine uptake. Our findings may be relevant to people seeking to address vaccine hesitancy [39], and in addressing catch-up campaigns with existing vaccines, in the context of disease outbreaks e.g. against Measles, or novel campaigns for emergency threats.

### Limitations of this study

We used a local population health management dataset including nearly 1 million people and data linked across primary, secondary, community, and mental health care. This is a nearly complete coverage of the area. We did not impute missing data for primary language or ethnicity and included these people in the analysis in their ‘missing’ categories. There was substantial missing information for primary language (66%) and ethnicity (20%). Nobody over the age of 57 years had a recorded primary language, whereas this was missing in 53% aged 57 years or less. Ethnicity was more likely to be missing (28%) amongst younger people (under 35 years) than citizens aged > 35 years (15%). We therefore decided not to match exposed and unexposed on primary language and kept people with missing ethnicity as a separate sub-group in analyses. To the extent that primary language influences vaccine uptake and is independent of other matching variables, and missingness in ethnicity relates to different ethnicities between exposed and unexposed areas, this may add to noise and attenuate our comparison of vaccine uptake between groups. We also did not have information about occupation (e.g., health, social, and residential care workers), which probably lead to some misallocation of people to JCVI priority groups for vaccine eligibility dates. Since occupations in health and social care would make someone eligible when they would not otherwise be, this would likely have the effect of someone appearing to have the vaccine earlier than they would otherwise be eligible. In areas where a large proportion of the community works in health or social care, this could result in the reduction of the apparent impact of a community vaccination intervention. We saw large heterogeneity in our results between outreach clinics. This could be the result of clinics being initiated on different dates during the pandemic period, with varying background vaccination rates over time; variation in the implementation and marketing of the clinics; clinics taking place on one versus multiple dates; or other unmeasured factors.

Trends in vaccination before outreach clinic index dates in intervention and control groups were not always parallel, which could have an impact on the results. In most cases this was due to a shallower pre-intervention trend in the exposed areas compared to the control areas, which would bias our results in favour of finding a difference since there is more scope for the rate of change to increase in the intervention group. However, the effect of any bias is likely to be minimal since the biggest difference between exposed and control groups is 0.09% vaccinated per day in the period before the outreach clinic.

It is possible that our definition of exposure, which was based on anyone resident within one mile of local vaccine clinics, is too broad. It is also difficult to define truly ‘unexposed’ citizens. During the pandemic there were several options available for people to receive a first vaccine, including walk-in settings, their own GP surgery, and pharmacies, these may have diluted the effect of outreach clinics.

We did not directly compare effects for ethnic minority groups to white groups, since we were concerned that this would make our analyses more complex and difficult to interpret. However, stratified analyses showed no effect in either ethnic group, and a large overlap between their confidence intervals. Outreach clinic sites were selected to be accessible to populations of ethnic minority, and were developed in partnership with communities, but were not restricted to Citizens of ethnic minority since vaccine eligibility was decided by nationally determined criteria.

The location of outreach was not random and was chosen based on need, therefore despite matching people living close to an outreach clinic may systematically differ to those who live further away. For example, people living close to an outreach clinic were more often young than those living further away, and could have different types of occupation (on which we had no data). This may mean that fewer people living near an outreach clinic were eligible for a vaccine at the same time as those living further away, even despite our efforts to match on age and vaccine eligibility.

Our study is quantitative and cannot capture any qualitative benefits (e.g. peer-to-peer socialising and vaccine advocacy) of outreach communities on the target communities. We also do not have the data to conduct an economic analysis of the cost-effectiveness of outreach vaccination clinics, but given the apparently negative results this is less crucial. It is possible that outreach clinics helped to maintain vaccine uptake and that without them there would have been bigger differences in uptake.

Outreach clinics comprise a complex intervention and potentially address several barriers to accessing vaccines, such as tailored messaging, reduced travel barriers, a sense of a community being valued enough to provide local services, and a sense of belonging as people communally attend their local setting. In this study we only examined one aspect of these outreach clinics, namely geographic distance, and our findings suggest that addressing geographic or travel distance from a vaccine clinic alone is unlikely to be sufficient in improving vaccine uptake.

### What this study adds

High vaccine uptake has benefits for society and for individuals [40], and so efforts will continue to maximise uptake, particularly in groups who are at higher risk of complications. We did not find evidence to support an effect of outreach vaccine clinics on COVID-19 primary immunisation, beyond the doses delivered directly in the community vaccination clinics. During the primary COVID-19 vaccination campaign there were several options available for people to access vaccination, e.g., mass vaccination centres, GP surgeries, and pharmacies, and this will limit our power to detect an effect.

Further work should investigate the relative importance of (among other things) distance, transport, language, and culturally safe spaces as factors determining choices to receive an offer of a vaccine.

## Conclusion

We found that people living within one mile of a community-based vaccine clinic, which were set up to increase COVID 19 vaccine uptake in groups traditionally known to show greater vaccine hesitancy, were not more likely to have a first COVID-19 vaccine within 6 weeks of the clinic than those living further away.

### Electronic supplementary material

Below is the link to the electronic supplementary material.

Supplementary Material 1

Supplementary Material 2

## Data Availability

This study is based on pseudonymised data from the BNSSG system-wide dataset obtained under an approved data sharing agreement between University of Bristol and NHS Bristol, North Somerset, and South Gloucestershire CCG (2021-7764). The data are provided by patients and collected by the NHS as part of their care and support. Access requests would have to be made to BNSSG CCG.

## Declarations

### Author Statement

This publication is the work of the authors, who serve as guarantors for the contents of this paper. SC and CK conceptualised the study. TJ contributed to study design, performed data cleaning and analysis and wrote the manuscript. SC, MTR, KT, YBS and FdV contributed to study design, analysis and interpretation of results. TJ had full access to the data in the study and takes responsibility for the integrity of the data and the accuracy of the data analysis. All authors contributed to the writing of the manuscript.

### Data sharing

This study is based on pseudonymised data from the BNSSG system-wide dataset obtained under an approved data sharing agreement between University of Bristol and NHS Bristol, North Somerset, and South Gloucestershire CCG (2021–7764). The data are provided by patients and collected by the NHS as part of their care and support. Access requests would have to be made to BNSSG CCG.

### Transparency

The manuscript’s guarantor (TJ) affirms that the manuscript is an honest, accurate, and transparent account of the study being reported; that no important aspects of the study have been omitted; and that any discrepancies from the study as originally planned have been explained.
