# Supplementary material for: The impact of targeted local outreach clinics to improve COVID-19 vaccine uptake: controlled interrupted time series in South West England
Source: Arch Public Health. 2024 Aug 7;82:118. doi: 10.1186/s13690-024-01341-1 (PMC11304932; doi:10.1186/s13690-024-01341-1)
Supplement: Supplementary file 2 — Supplementary Material 2 [file 13690_2024_1341_MOESM2_ESM.pdf]

Supplementary Figure F1: The impact of local COVID-19 vaccine outreach clinics on cumulative vaccination at 6 weeks post-intervention compared to matched controls, by ethnic group, limited to subset of clinics most likely to focus on global majority groups.

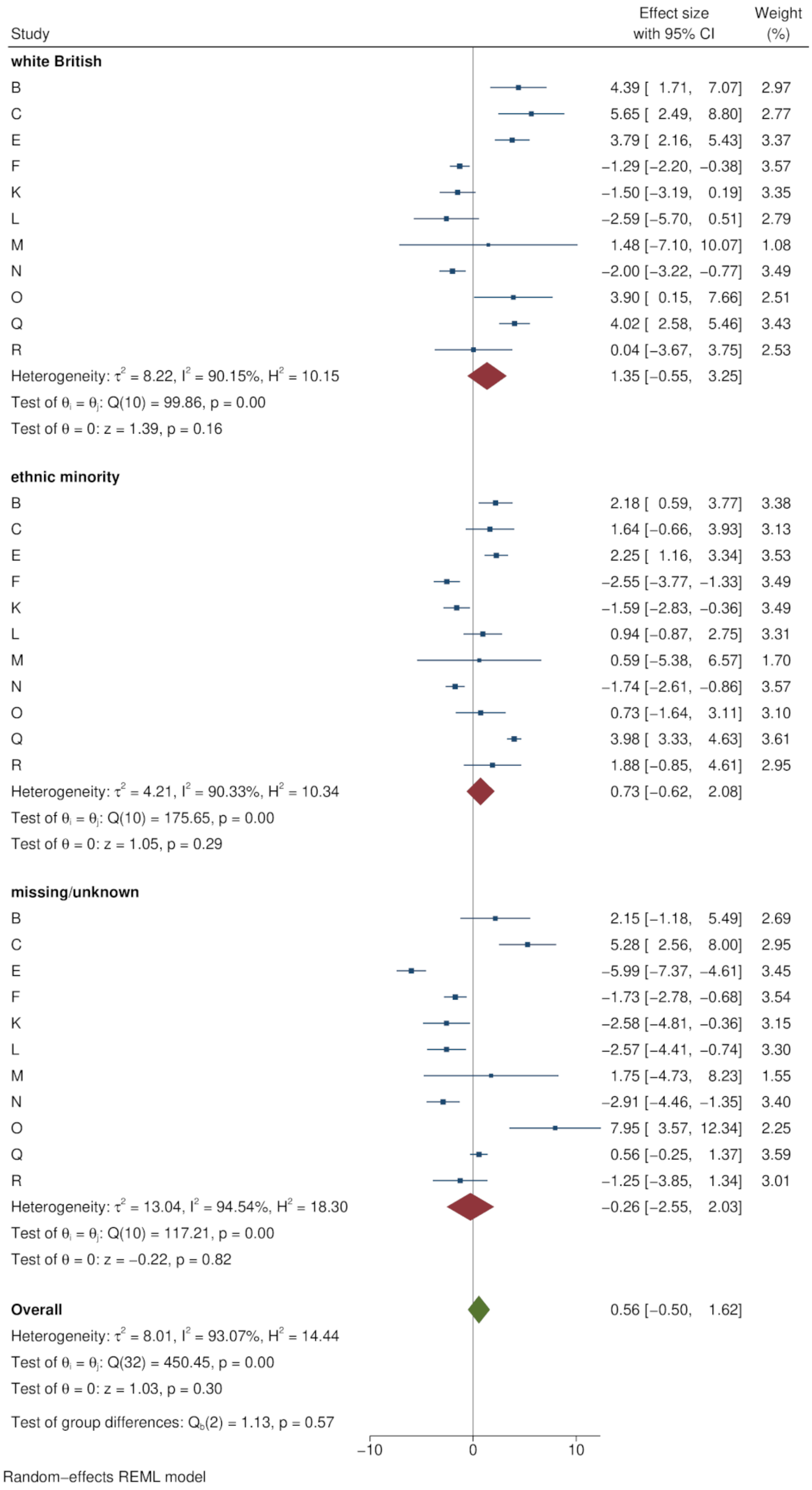

Supplementary Figure F2: The impact of local COVID-19 vaccine outreach clinics on cumulative vaccination (%) at 6 weeks post-intervention compared to matched controls, by age group, limited to subset of clinics most likely to focus on global majority groups.

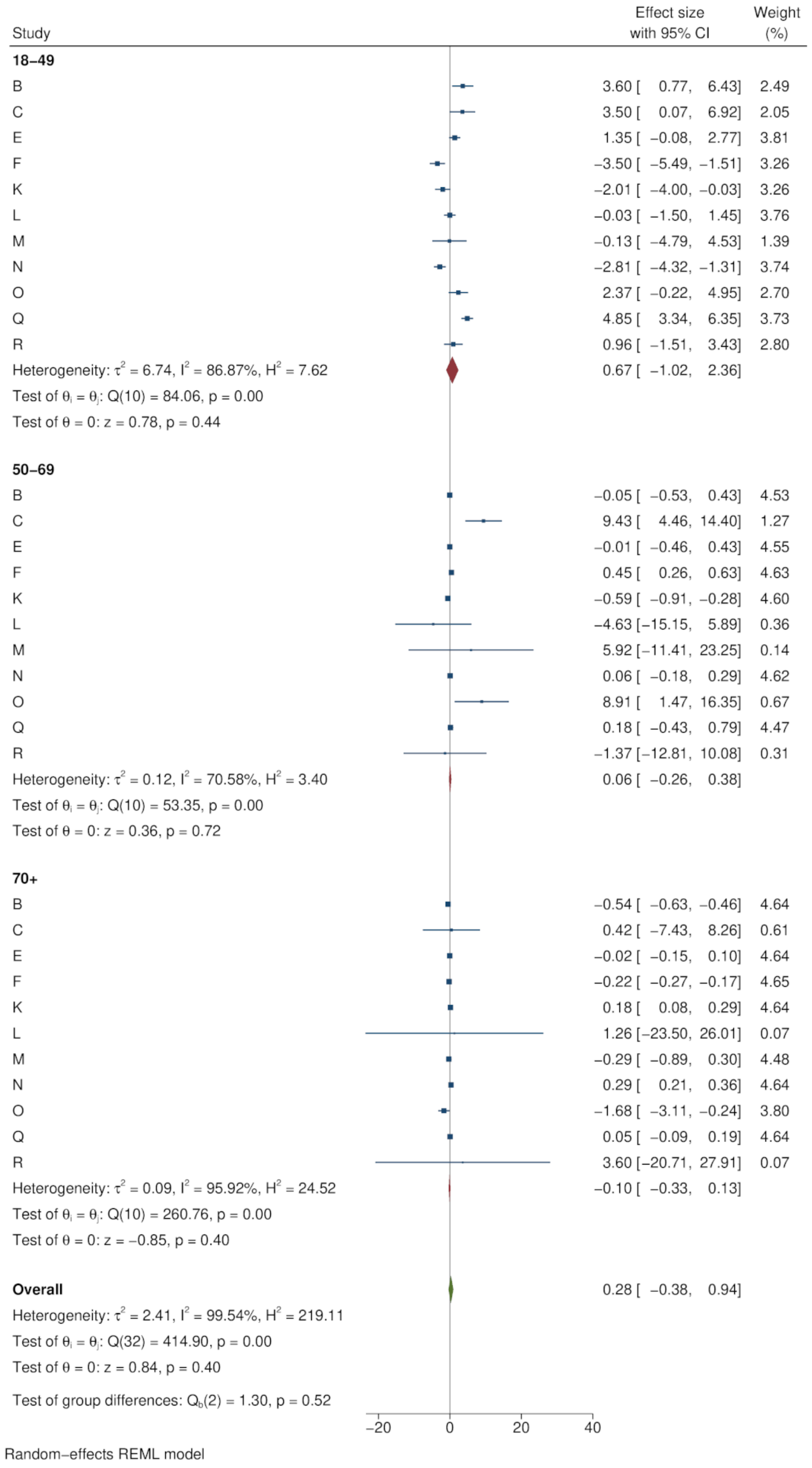

Supplementary Figure F3: The impact of local COVID-19 vaccine outreach clinics on cumulative vaccination (%) at 6 weeks post-intervention compared to matched controls, by deprivation group, limited to subset of clinics most likely to focus on global majority groups.

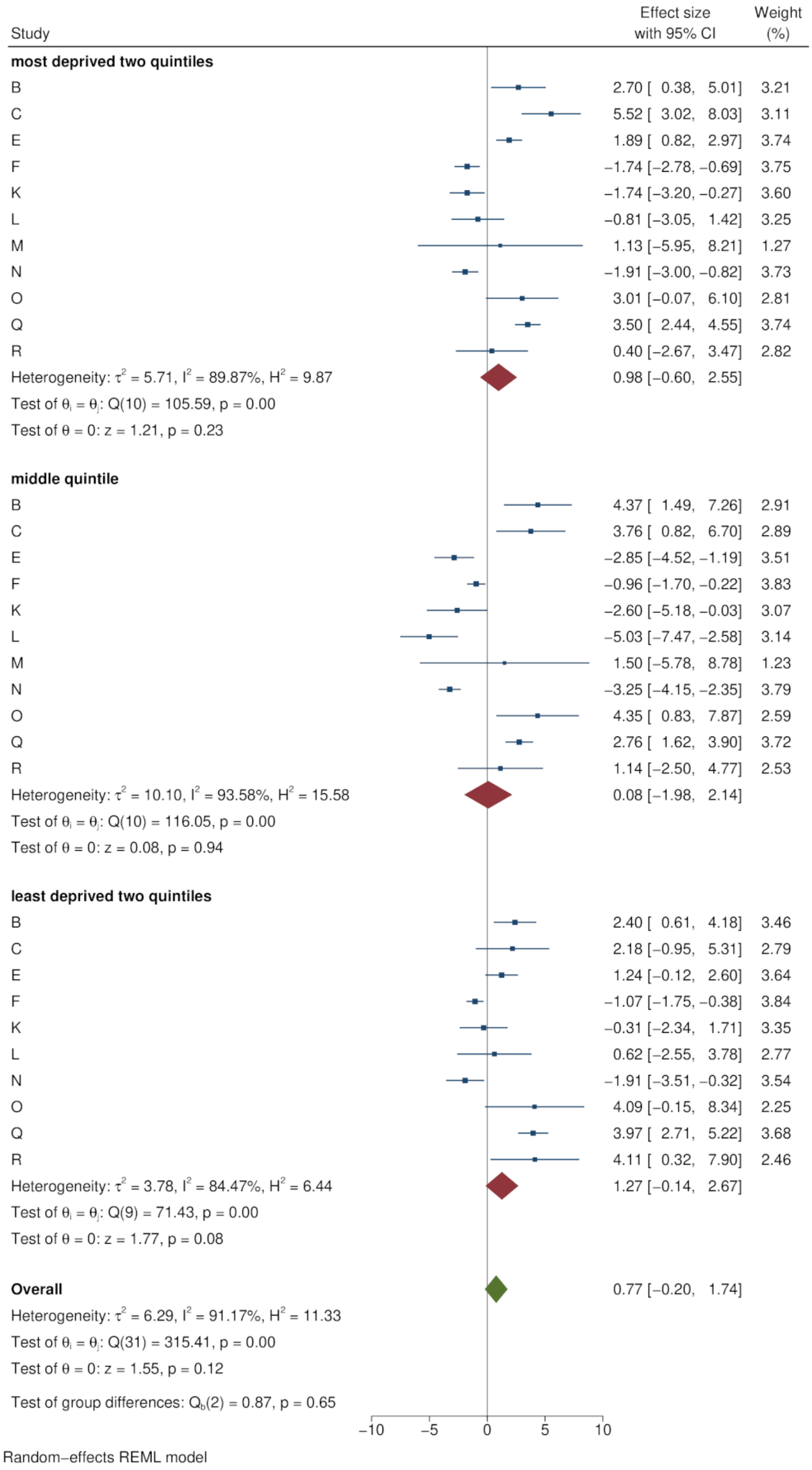

Supplementary Figure F4: Cumulative COVID-19 vaccine uptake amongst people within 1 mile of the area A intervention and matched controls

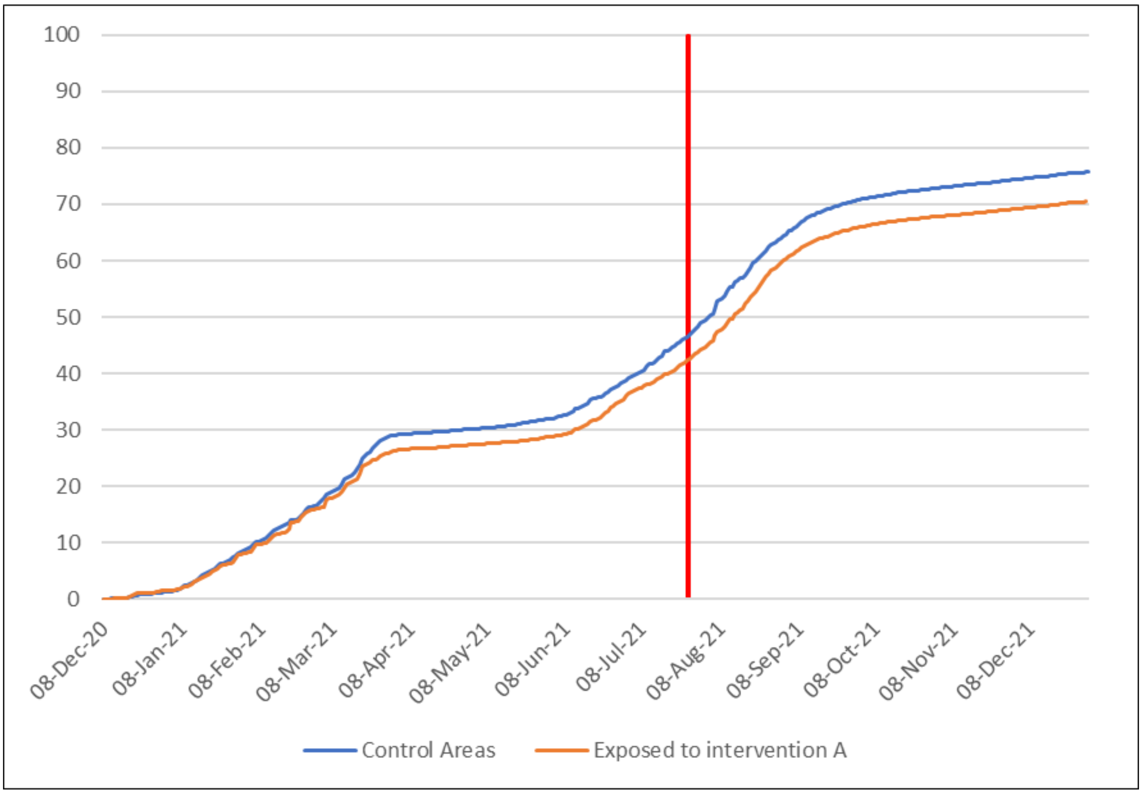

Supplementary Figure F5: Cumulative COVID-19 vaccine uptake amongst people within 1 mile of the area C intervention and matched controls

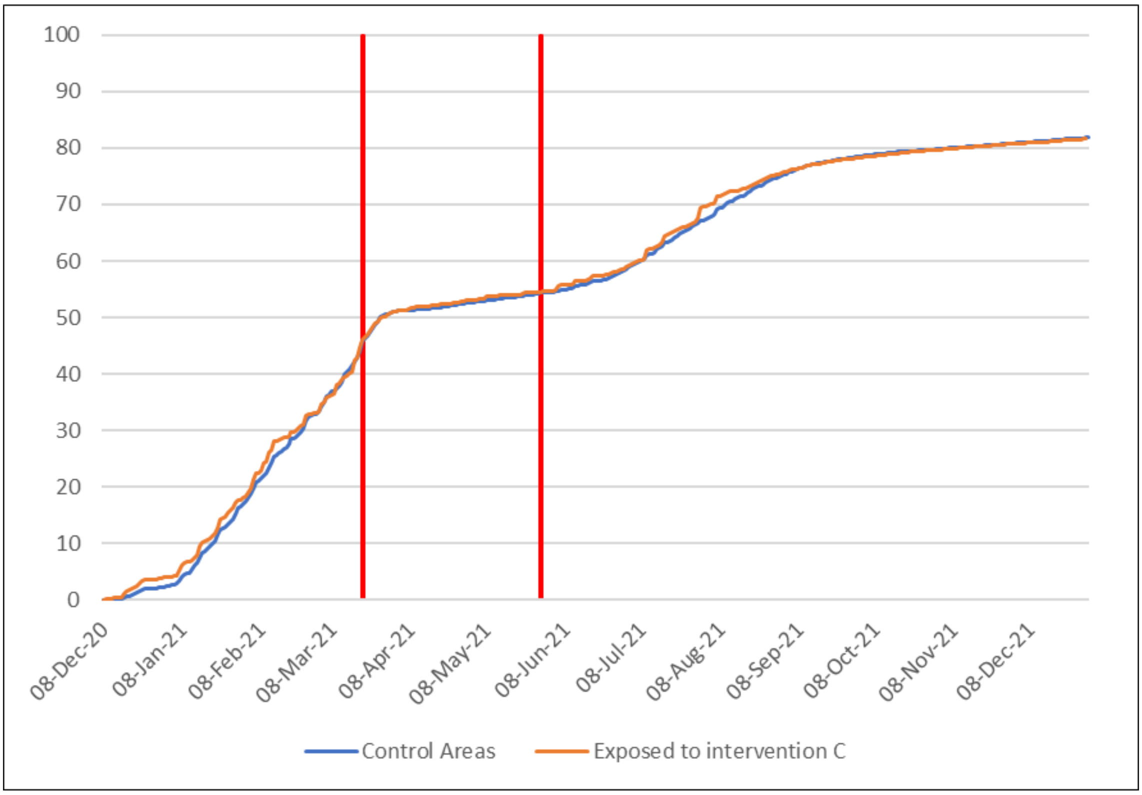

Supplementary Figure F6: Cumulative COVID-19 vaccine uptake amongst people within 1 mile of the area D intervention and matched controls

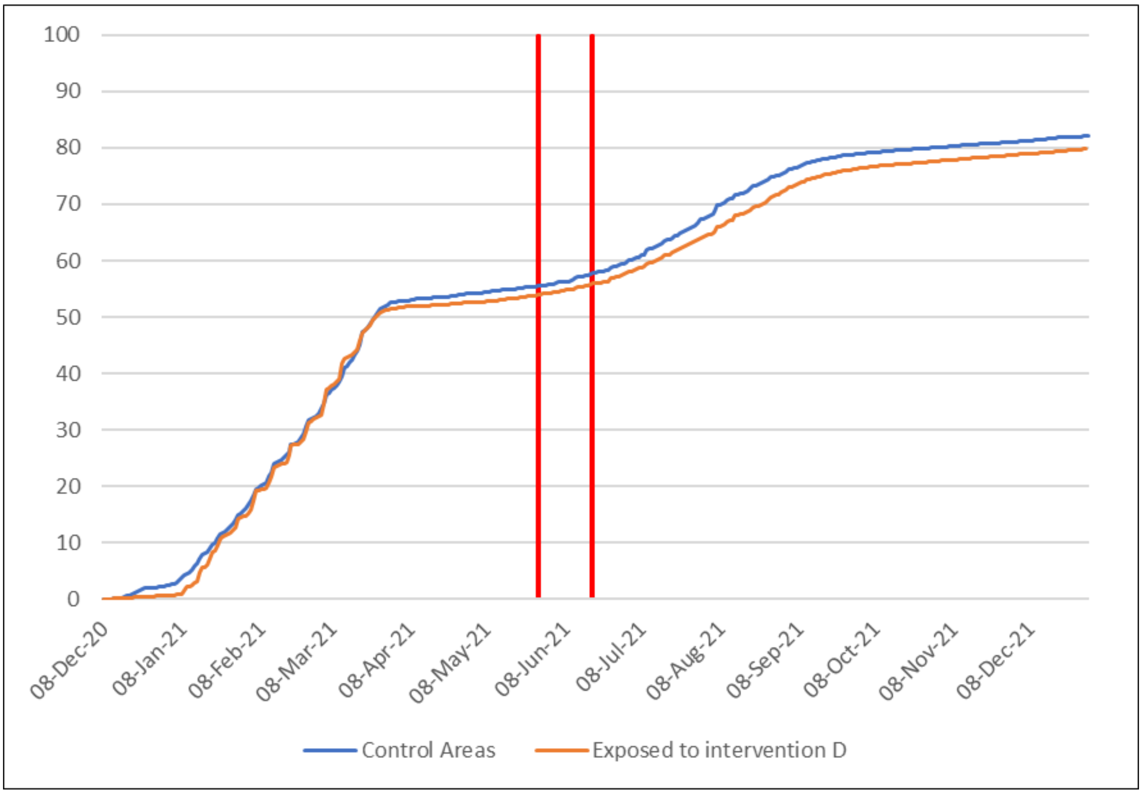

Supplementary Figure F7: Cumulative COVID-19 vaccine uptake amongst people within 1 mile of the area E intervention and matched controls

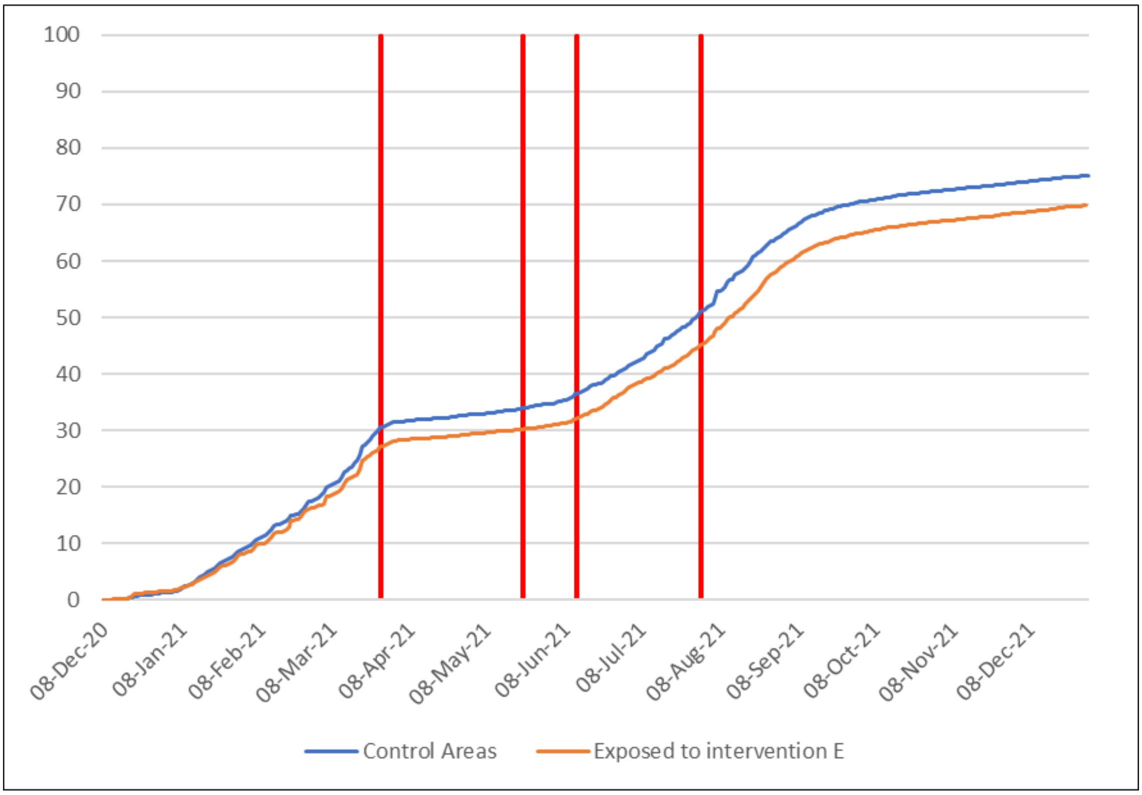

Supplementary Figure F8: Cumulative COVID-19 vaccine uptake amongst people within 1 mile of the area F intervention and matched controls

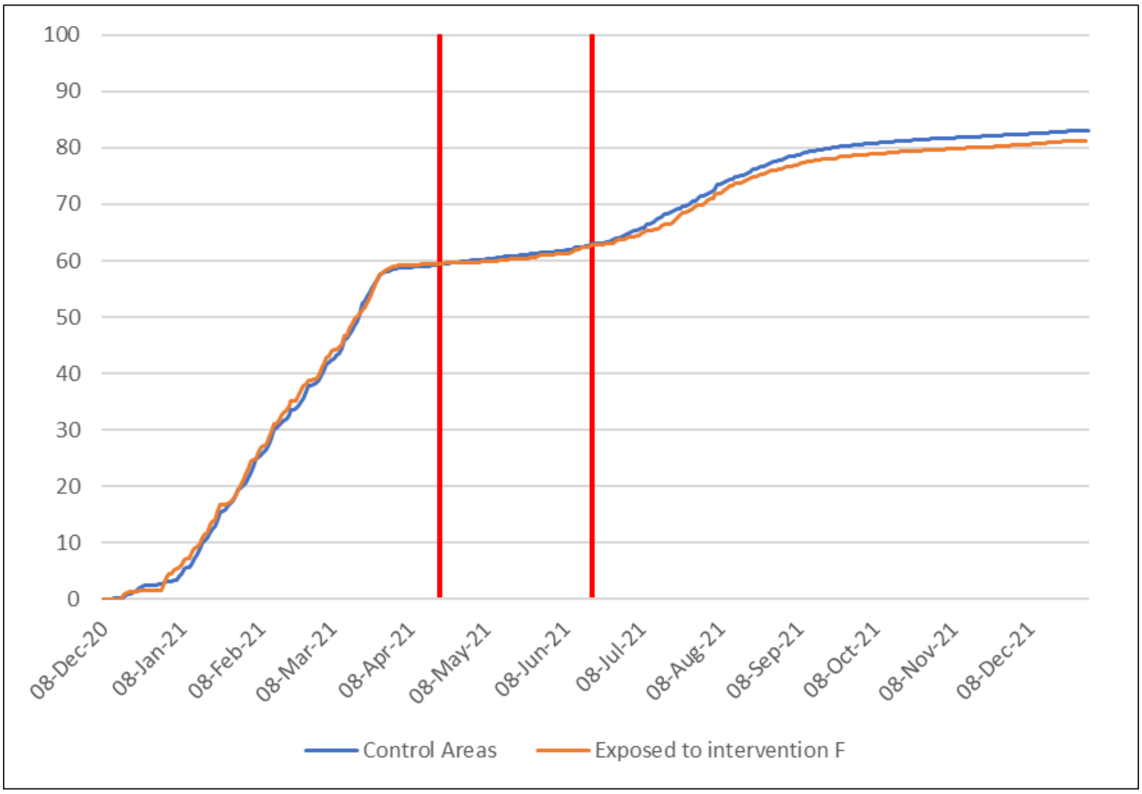

Supplementary Figure F9: Cumulative COVID-19 vaccine uptake amongst people within 1 mile of the area G intervention and matched controls

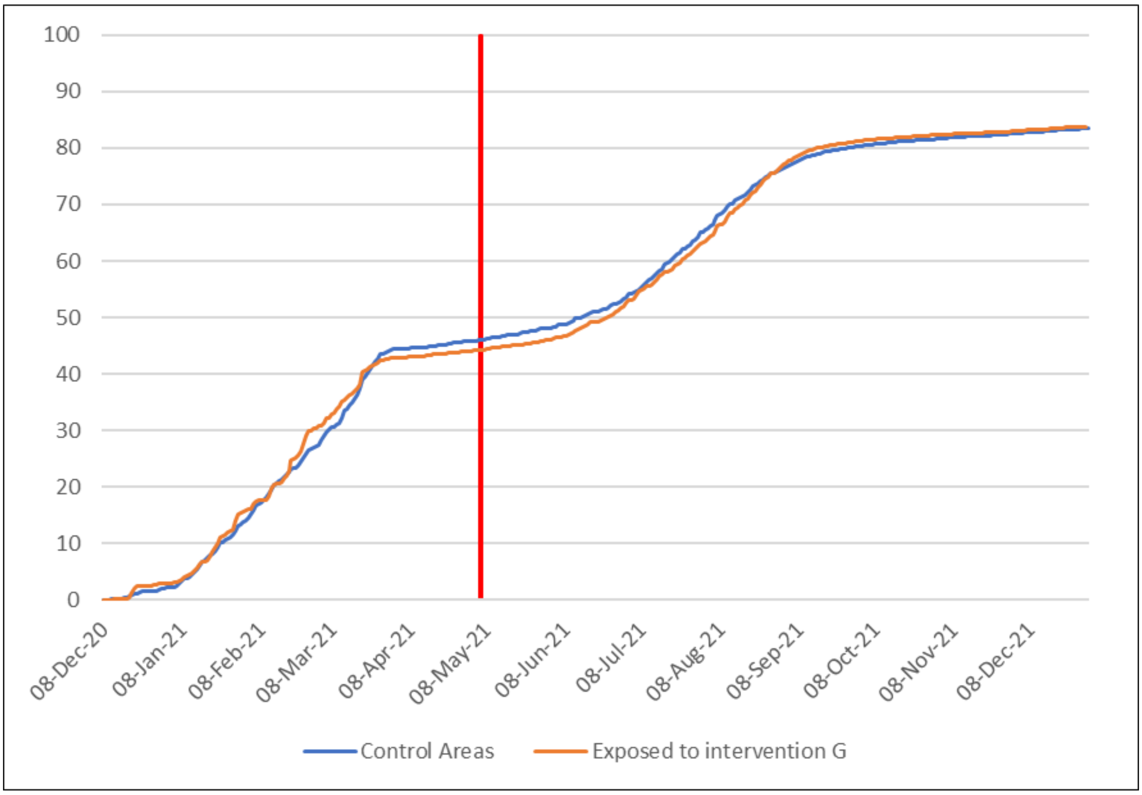

Supplementary Figure 10: Cumulative COVID-19 vaccine uptake amongst people within 1 mile of the area H intervention and matched controls

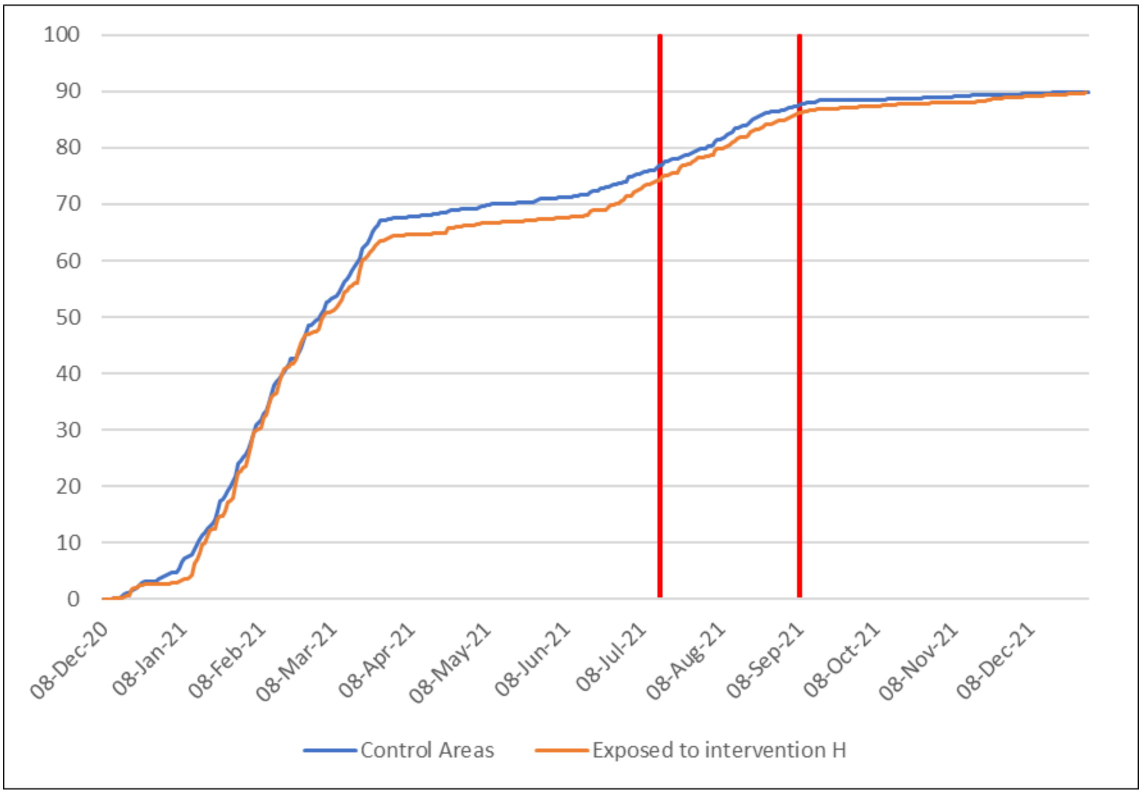

Supplementary Figure 11: Cumulative COVID-19 vaccine uptake amongst people within 1 mile of the area I intervention and matched controls

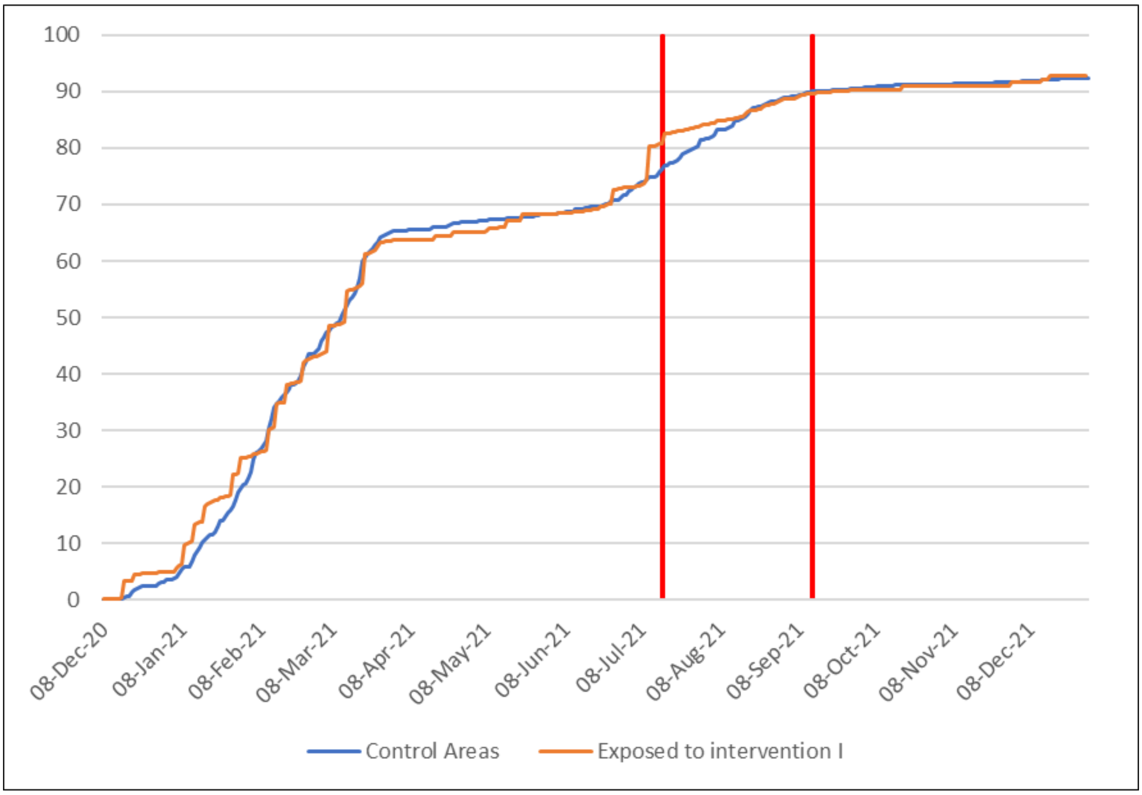

Supplementary Figure 12: Cumulative COVID-19 vaccine uptake amongst people within 1 mile of the area J intervention and matched controls

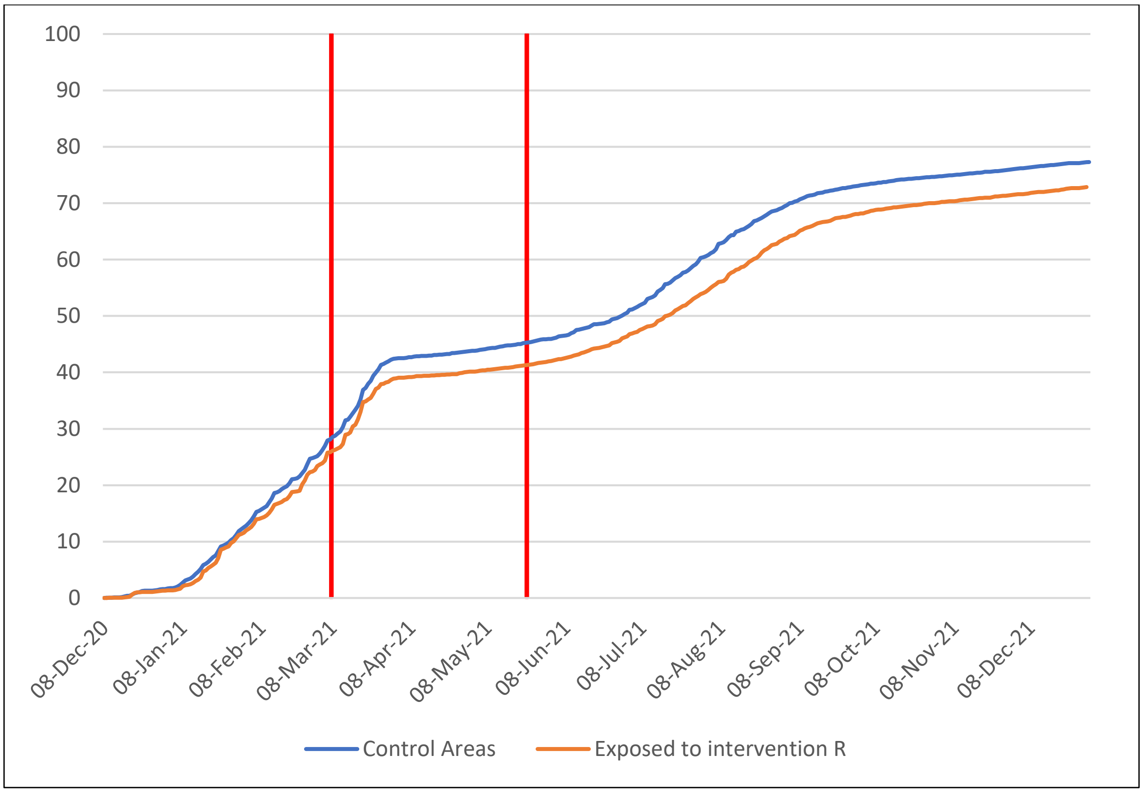

Supplementary Figure 13: Cumulative COVID-19 vaccine uptake amongst people within 1 mile of the area K intervention and matched controls

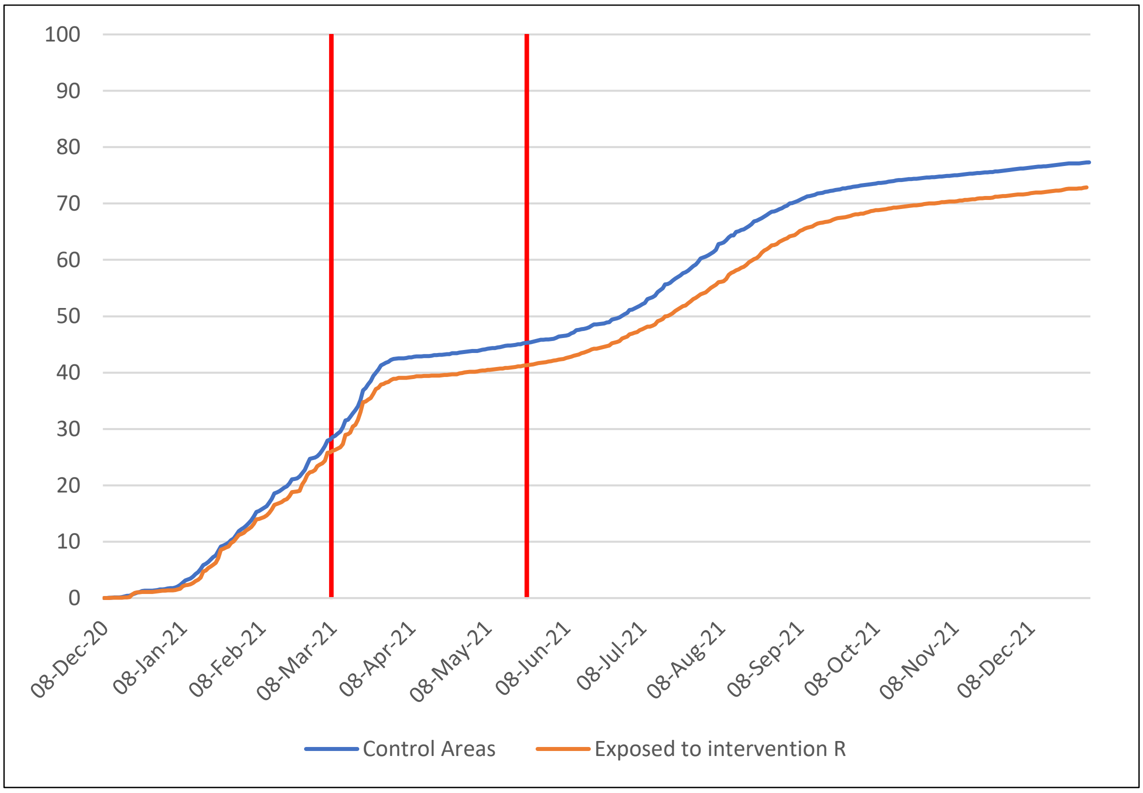

Supplementary Figure 14: Cumulative COVID-19 vaccine uptake amongst people within 1 mile of the area L intervention and matched controls

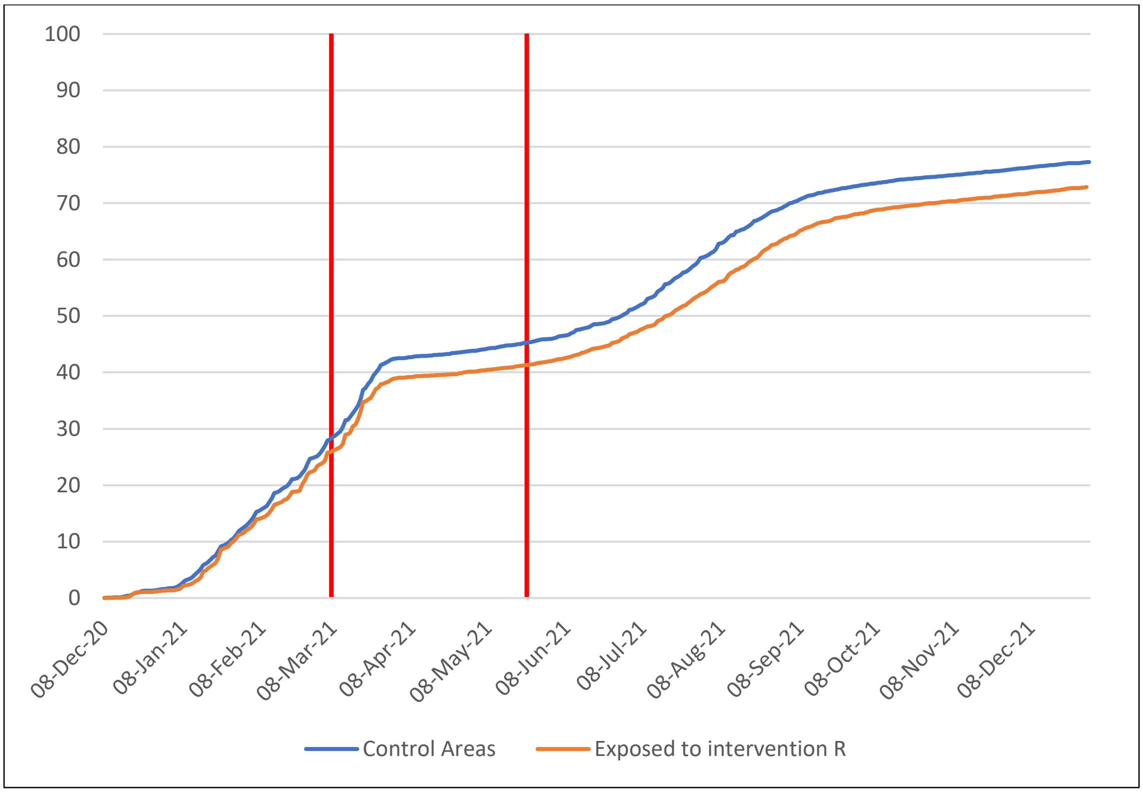

Supplementary Figure 15: Cumulative COVID-19 vaccine uptake amongst people within 1 mile of the area M intervention and matched controls

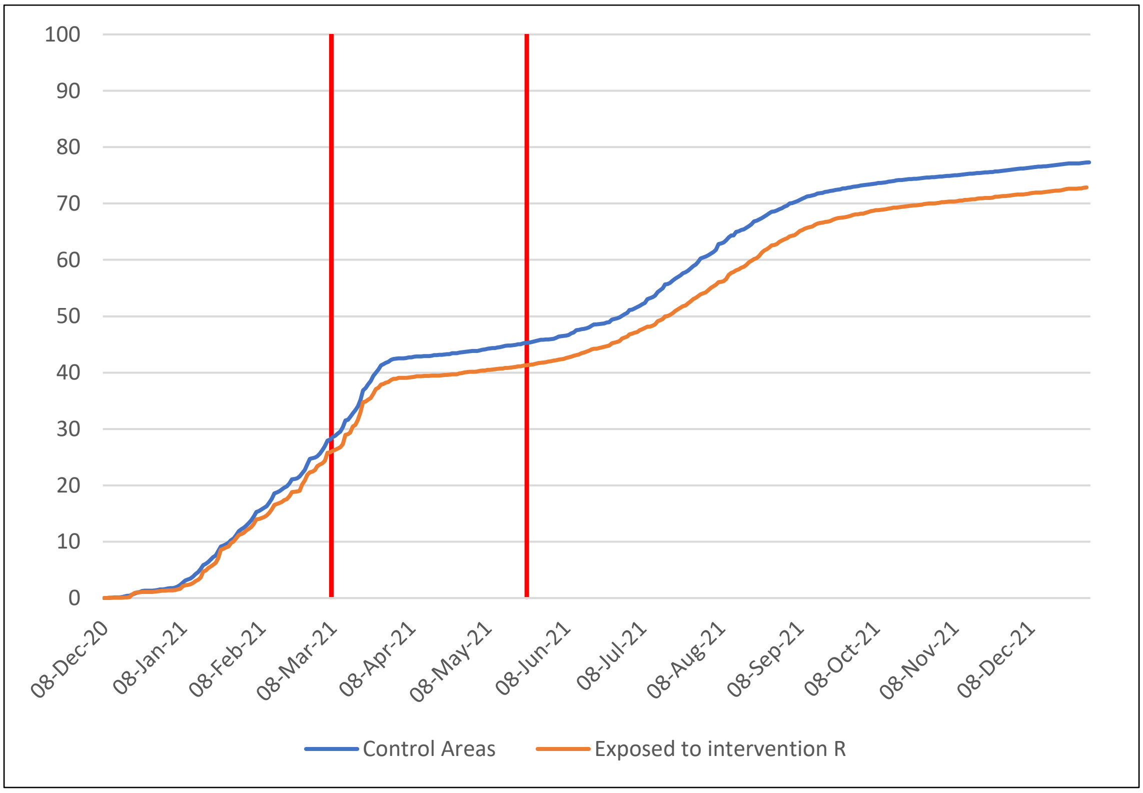

Supplementary Figure 16: Cumulative COVID-19 vaccine uptake amongst people within 1 mile of the area N intervention and matched controls

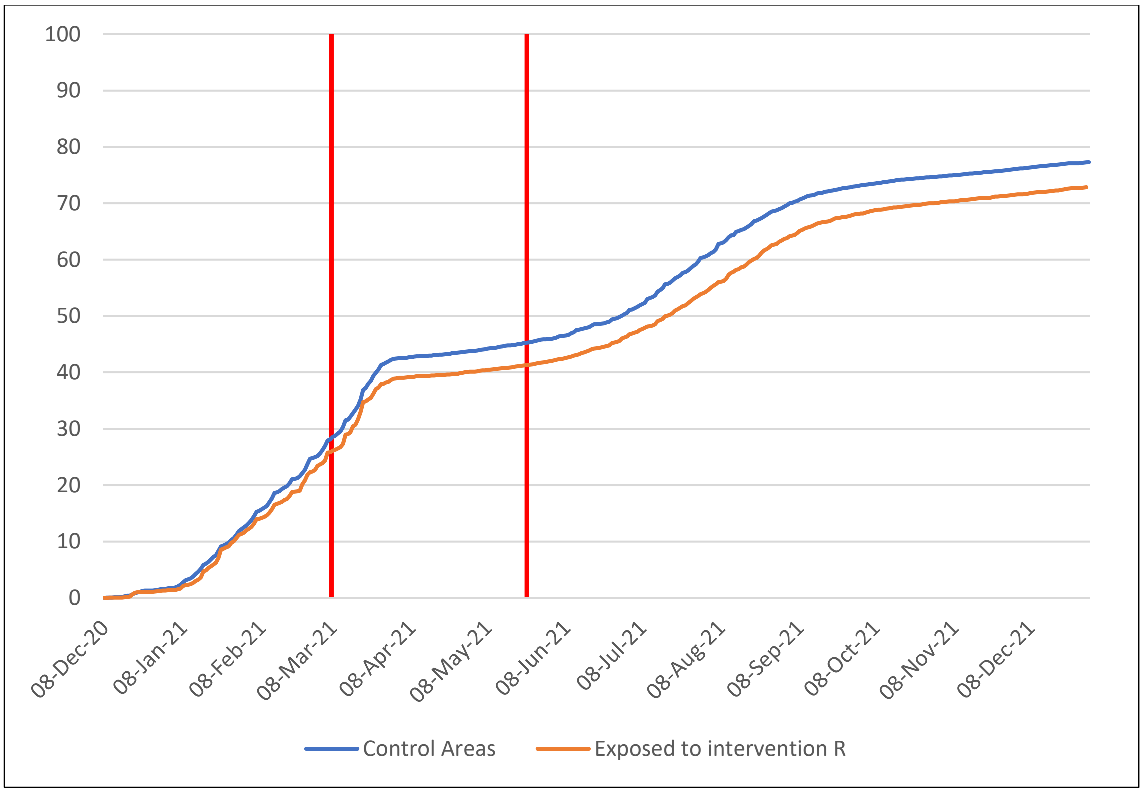

Supplementary Figure 17: Cumulative COVID-19 vaccine uptake amongst people within 1 mile of the area O intervention and matched controls

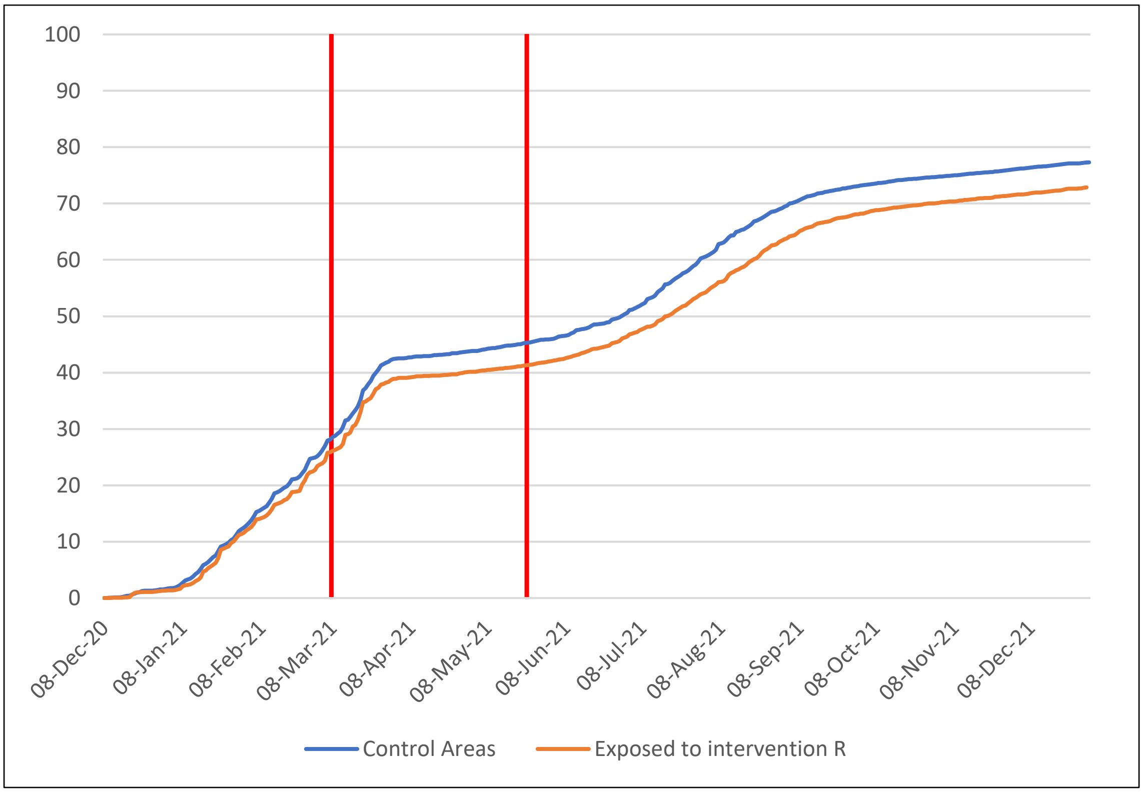

Supplementary Figure 18: Cumulative COVID-19 vaccine uptake amongst people within 1 mile of the area P intervention and matched controls

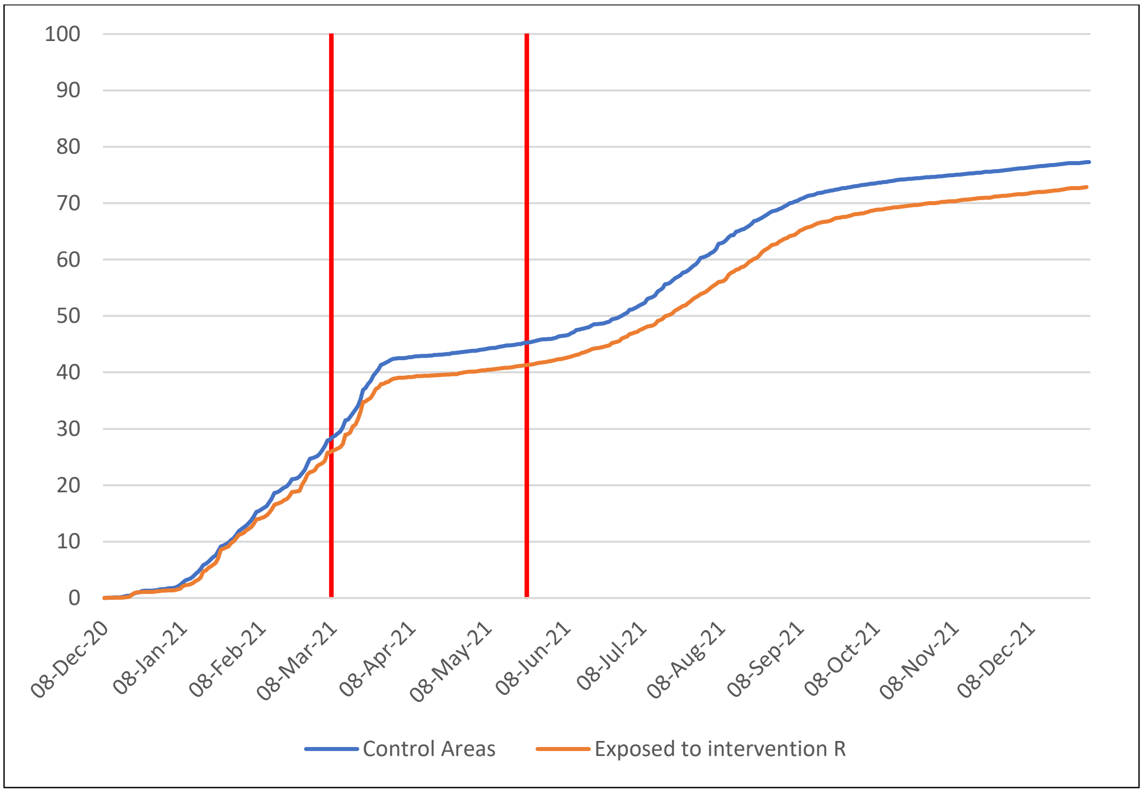

Supplementary Figure 19: Cumulative COVID-19 vaccine uptake amongst people within 1 mile of the area Q intervention and matched controls

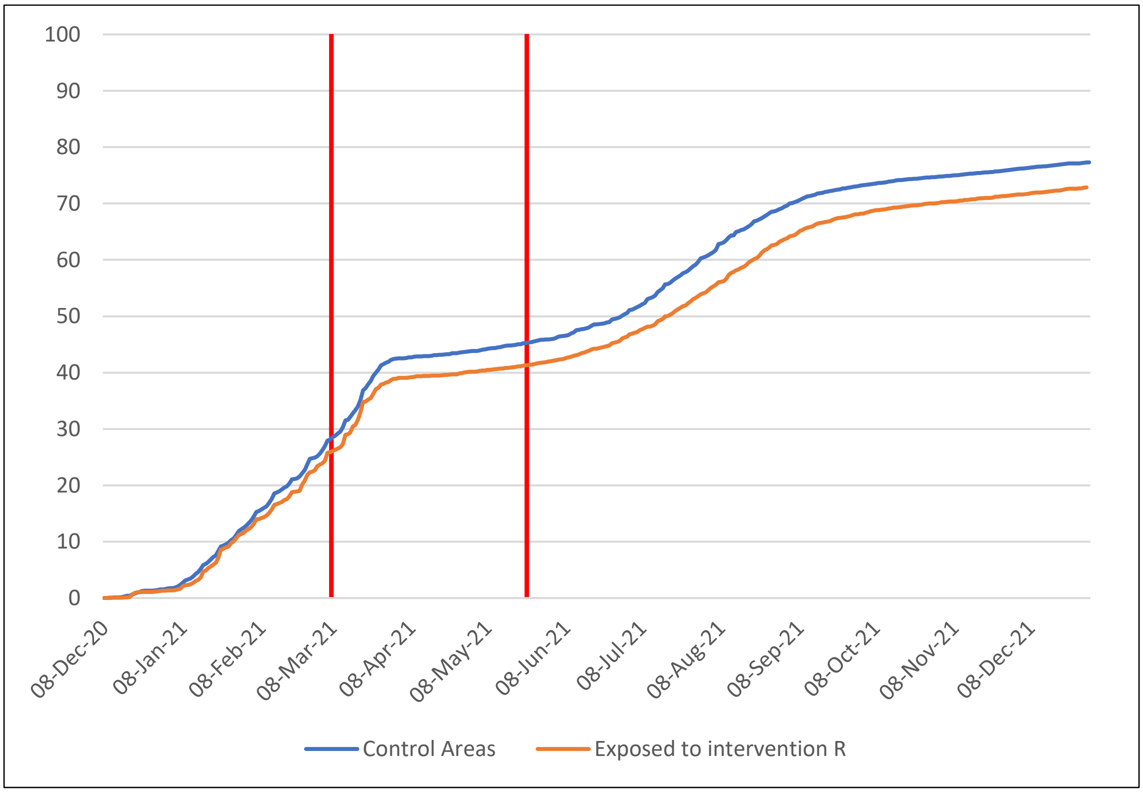

Supplementary Figure 20: Cumulative COVID-19 vaccine uptake amongst people within 1 mile of the area R intervention and matched controls

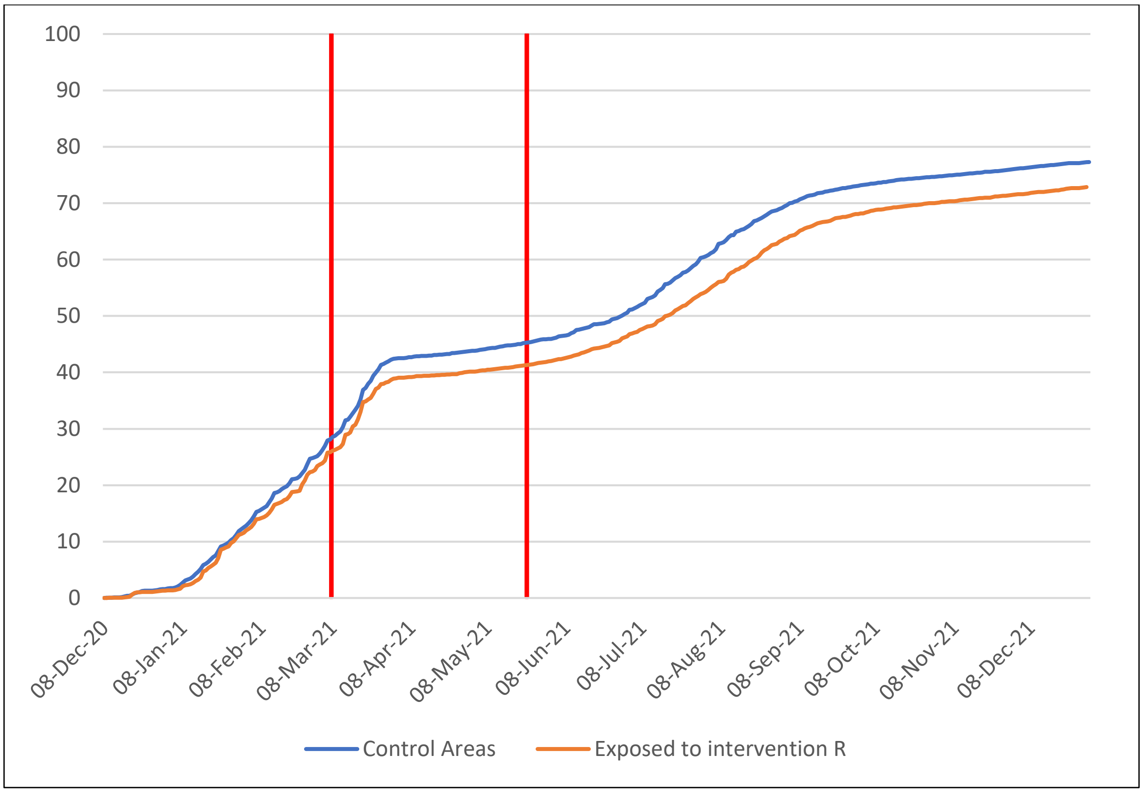

Supplementary Table T1: Categorising textual labels of ethnicity into ethnic groups

Ethnic Group	Ethnicity
bangladeshi	asian or asian british: bangladeshi - england and wales ethnic category 2011 census
	asian or asian british: bangladeshi - northern ireland ethnic category 2011 census
	bangladeshi
	bangladeshi or british bangladeshi - ethnic category 2001 census
	race: bangladeshi
black african	african: african african scottish or african british - scotland ethnic category 2011 census
	african: any other african - scotland ethnic category 2011 census
	african - ethnic category 2001 census
	black - other african country
	black african
	black north african
	black or african or caribbean or black british: african - england and wales ethnic category 2011 census
	black or african or caribbean or black british: african - northern ireland ethnic category 2011 census
	nigerian - ethnic category 2001 census
	somali - ethnic category 2001 census
black caribbean	afro-caribbean
	black caribbean
	black caribbean/w.i./guyana
	black or african or caribbean or black british: caribbean - england and wales ethnic category 2011 census
	black or african or caribbean or black british: caribbean - northern ireland ethnic category 2011 census
	black west indian
	caribbean - ethnic category 2001 census
	caribbean i./w.i./guyana (nmo)

	caribbean or black: caribbean caribbean scottish or caribbean british - scotland ethnic category 2011 census
	race: west indian
black other	black - ethnic group
	black - other asian
	black black - other
	black british
	black british - ethnic category 2001 census
	black east african asian/indo-caribbean
	black n african/arab/iranian
	black or african or caribbean or black british: other black or african or caribbean background - england and wales ethnic category 2011 census
	black or african or caribbean or black british: other black or african or caribbean background - northern ireland ethnic category 2011 census
	black other non-mixed origin
	caribbean or black: any other black or caribbean group - scotland ethnic category 2011 census
	other black - black/asian orig
	other black background - ethnic category 2001 census
	other black ethnic group
	other black or black unspecified - ethnic category 2001 census
chinese	asian and chinese - ethnic category 2001 census
	asian or asian british: chinese - england and wales ethnic category 2011 census
	asian or asian british: chinese - northern ireland ethnic category 2011 census
	black and chinese - ethnic category 2001 census
	chinese
	chinese - ethnic category 2001 census
	race: chinese
gypsa/roma/traveller	gypsies
	gypsy/romany - ethnic category 2001 census
	irish traveller

	irish traveller - ethnic category 2001 census
	polish roma
	traveller - ethnic category 2001 census
indian	asian or asian british: indian - england and wales ethnic category 2011 census
	asian or asian british: indian - northern ireland ethnic category 2011 census
	indian
	indian or british indian - ethnic category 2001 census
Missing / Not recorded	ethnic category not stated - 2001 census
	ethnic group not recorded
	NULL
	refusal by patient to provide information about ethnic group
mixed	afro-caucasian
	black - other mixed
	black african and white
	black and asian - ethnic category 2001 census
	black and white - ethnic category 2001 census
	black caribbean and white
	black east african asian
	caribbean asian - ethnic category 2001 census
	chinese and white - ethnic category 2001 census
	east african asian (nmo)
	mixed asian - ethnic category 2001 census
	mixed black - ethnic category 2001 census
	mixed ethnic census group
	mixed multiple ethnic groups: any other mixed or multiple ethnic background - england and wales ethnic category 2011 census
	mixed multiple ethnic groups: any other mixed or multiple ethnic background - northern ireland ethnic category 2011 census
	mixed multiple ethnic groups: white and asian - england and wales ethnic category 2011 census

	mixed multiple ethnic groups: white and asian - northern ireland ethnic category 2011 census
	mixed multiple ethnic groups: white and black african - england and wales ethnic category 2011 census
	mixed multiple ethnic groups: white and black african - northern ireland ethnic category 2011 census
	mixed multiple ethnic groups: white and black caribbean - england and wales ethnic category 2011 census
	mixed multiple ethnic groups: white and black caribbean - northern ireland ethnic category 2011 census
	mixed racial group
	other black - black/white orig
	other ethnic asian/white orig
	other ethnic black/white orig
	other ethnic mixed origin
	other ethnic mixed white orig
	other ethnic other mixed orig
	other mixed background - ethnic category 2001 census
	other mixed or mixed unspecified - ethnic category 2001 census
	other mixed white - ethnic category 2001 census
	white and asian - ethnic category 2001 census
	white and black african - ethnic category 2001 census
	white and black caribbean - ethnic category 2001 census
other	any other group - ethnic category 2001 census
	arab - ethnic category 2001 census
	arabs
	black indian sub-continent
	brazilian
	brit. ethnic minor. spec.(nmo)
	brit. ethnic minor. unsp (nmo)
	buddhist - ethnic category 2001 census
	commonwealth of (russian) independent states - ethnic category 2001 census

	cypriot (part not stated) - ethnic category 2001 census
	czech
	e afric asian/indo-carib (nmo)
	east african asian - ethnic category 2001 census
	ethnic category - 2001 census
	ethnic category - 2011 census
	ethnic category - 2011 census england and wales
	ethnic group
	ethnic group finding
	ethnic groups (census) nos
	ethnicity and other related nationality data
	filipino - ethnic category 2001 census
	greek - ethnic category 2001 census
	greek (nmo)
	greek cypriot - ethnic category 2001 census
	greek cypriot (nmo)
	greek/greek cypriot (nmo)
	hindu - ethnic category 2001 census
	iranian - ethnic category 2001 census
	iranian (nmo)
	israeli - ethnic category 2001 census
	jewish - ethnic category 2001 census
	koreans
	kurdish - ethnic category 2001 census
	latin american - ethnic category 2001 census
	middle eastern (excluding israeli iranian and arab) - ethnic category 2001 census
	moroccan - ethnic category 2001 census
	multi-ethnic islands: mauritian or seychellois or maldivian or st helena - ethnic category 2001 census
	muslim - ethnic category 2001 census

	n african arab/iranian (nmo)
	nepali
	new zealand ethnic group nos
	new zealand ethnic groups
	new zealand maori
	north african - ethnic category 2001 census
	other - ethnic category 2001 census
	other african countries (nmo)
	other ethnic group: any other ethnic group - england and wales ethnic category 2011 census
	other ethnic group: any other ethnic group - northern ireland ethnic category 2011 census
	other ethnic nec (nmo)
	other ethnic non-mixed (nmo)
	other republics which made up the former yugoslavia - ethnic category 2001 census
	punjabi - ethnic category 2001 census
	sikh - ethnic category 2001 census
	slovak
	south and central american - ethnic category 2001 census
	tamil - ethnic category 2001 census
	turkish - ethnic category 2001 census
	turkish (nmo)
	turkish cypriot - ethnic category 2001 census
	turkish/turkish cypriot (nmo)
	yemeni
other asian	asian - ethnic group
	asian or asian british: any other asian background - england and wales ethnic category 2011 census
	asian or asian scottish or asian british: any other asian group - scotland ethnic category 2011 census

	asian or asian scottish or asian british: indian indian scottish or indian british - scotland ethnic category 2011 census
	asian or asian scottish or asian british: pakistani pakistani scottish or pakistani british - scotland ethnic category 2011 census
	british asian - ethnic category 2001 census
	indian sub-continent (nmo)
	japanese
	japanese - ethnic category 2001 census
	kashmiri - ethnic category 2001 census
	malaysian - ethnic category 2001 census
	other asian (nmo)
	other asian background - ethnic category 2001 census
	other asian ethnic group
	other asian or asian unspecified - ethnic category 2001 census
	sinhalese - ethnic category 2001 census
	south east asian
	sri lankan - ethnic category 2001 census
	vietnamese
	vietnamese - ethnic category 2001 census
pakistani	asian or asian british: pakistani - england and wales ethnic category 2011 census
	asian or asian british: pakistani - northern ireland ethnic category 2011 census
	pakistani
	pakistani or british pakistani - ethnic category 2001 census
	race: pakistani
white british	british or mixed british - ethnic category 2001 census
	cornish - ethnic category 2001 census
	english - ethnic category 2001 census
	other white british ethnic group
	scottish - ethnic category 2001 census
	welsh - ethnic category 2001 census

	white british
	white british - ethnic category 2001 census
	white scottish
	white: english or welsh or scottish or northern irish or british - england and wales ethnic category 2011 census
	white: other british - scotland ethnic category 2011 census
	white: scottish - scotland ethnic category 2011 census
white irish	irish - ethnic category 2001 census
	irish (nmo)
	mixed irish and other white - ethnic category 2001 census
	northern irish - ethnic category 2001 census
	white irish
	white irish - ethnic category 2001 census
	white: irish - england and wales ethnic category 2011 census
white other	albanian - ethnic category 2001 census
	baltic states (estonian or latvian or lithuanian) - ethnic category 2001 census
	bosnian - ethnic category 2001 census
	bulgarian
	caucasian race
	croatian - ethnic category 2001 census
	italian - ethnic category 2001 census
	kosovan - ethnic category 2001 census
	other european (nmo)
	other white background - ethnic category 2001 census
	other white european or european unspecified or mixed european - ethnic category 2001 census
	other white or white unspecified - ethnic category 2001 census
	polish - ethnic category 2001 census
	portuguese
	race: white
	romanian

	serbian - ethnic category 2001 census
	white
	white - ethnic group
	white: any other white background - england and wales ethnic category 2011 census
	white: any other white ethnic group - scotland ethnic category 2011 census
	white: polish - scotland ethnic category 2011 census

Supplementary Table T2: Interventions targeting group 2: ethnic minorities, non-English speakers, refugees and asylum seekers

**Intervention ID**	**earliest date**	**latest date**	**interventions at this location**
A	26-Jul-21	26-Jul-21	1
B	20-Aug-21	20-Aug-21	1
C	20-Mar-21	29-May-21	2
D	28-May-21	18-Jun-21	2
E	27-Mar-21	31-Jul-21	4
F	19-Apr-21	18-Jun-21	2
G	05-May-21	05-May-21	1
H	15-Jul-21	08-Sep-21	2
I	16-Jul-21	13-Sep-21	2
J	09-Mar-21	16-Jun-21	5
K	30-Jun-21	08-Sep-21	11
L	27-Feb-21	15-May-21	3
M	24-Apr-21	24-Apr-21	1
N	29-Jun-21	14-Sep-21	11
O	31-Mar-21	31-Mar-21	1
P	19-Jun-21	19-Jun-21	1
Q	08-Aug-21	08-Aug-21	1
R	07-Mar-21	23-May-21	2

Supplementary Table T3a: Group 2 interventions that are within 1 mile of each other

**Group 2 Intervention 1**	**Group 2 Intervention 2 (within 1 mile)**
A	B E K
B	A E K
C	None within 1 mile
D	None within 1 mile
E	A B K L N Q
F	None within 1 mile
G	None within 1 mile
H	None within 1 mile
I	None within 1 mile
J	K L M N R
K	A B E J L M N O Q
L	E J K M N O P Q R
M	J K L N O P Q R
N	E J K L M O P Q R
O	K L M N P Q
P	L M N O Q
Q	E K L M N O P
R	J L M N

Supplementary Table T3b: Group 2 interventions that are within 1 mile of each other

**Group 2 Intervention 1**	**Group 2 Intervention 2 (within 1 mile)**																	
	A	B	C	D	E	F	G	H	I	J	K	L	M	N	O	P	Q	R
A																		
B																		
C																		
D																		
E																		
F																		
G																		
H																		
I																		
J																		
K																		

L																		
M																		
N																		
O																		
P																		
Q																		
R																		

Supplementary Table T4: The JCVI COVID-19 vaccination priority groups as defined in the JCVI 'green book'

Priority Group	Description
1	Residents in a care home for older adults
	Staff working in care homes for older adults
2	All aged 80+
	Frontline health and social care workers
3	All aged 75+
4	All aged 70+
	Clinically extremely vulnerable aged 16+
5	All aged 65+
6	At-risk adults aged 16-65
7	All aged 60+
8	All aged 55+
9	All aged 50+

Supplementary Statistical Methods

Cumulative vaccination uptake using Kaplan Meier methods

We considered follow-up for each person from the first day of the earliest monthly snapshot in which they appeared to the last day of the latest monthly snapshot in which they appeared, after which we censored them. We used Kaplan Meier methods [27] to calculate cumulative vaccination rates each day assuming non-informative censoring: each day a not-vaccinated rate was calculated out of those still 'at-risk '(not vaccinated) and present in the data (not censored). We calculated the cumulative not-vaccinated rate as the product of this daily rate and all previous daily rates, and we calculated the cumulative vaccinated percentage as one minus the cumulative not-vaccinated multiplied by 100.

Controlled interrupted time series analysis of cumulative vaccination at exposed and control areas

ITS analysis models the trend before an intervention and the trend after the intervention using segmented regression models, allowing for a change in trend on the intervention date [28,29]. We used a period of 6 weeks before and 6 weeks after the start of each local outreach clinic. Using segmented regression models, we predicted a 'counterfactual'; that is the increase in cumulative vaccine uptake at 6 weeks without the outreach clinic. We also estimated the increase in vaccine uptake at 6 weeks allowing for a change in trend on the outreach clinic date, based on the observed data in the post- outreach clinic period. The difference between these two estimates was considered as the impact of the outreach clinic on cumulative vaccine uptake at 6 weeks post-outreach clinic.

We additionally conducted the same analysis in the matched control group using the same index date, to account for any background changes in vaccination unrelated to the outreach clinic. Having estimated the impact on cumulative vaccination at 6 weeks after the index date in the outreach clinic area, and the equivalent impact in the control area (without the outreach clinic), we took the difference between these to estimate the impact of the outreach clinic.

We adjusted for serial autocorrelation in the models by allowing for an autocorrelation structure using Newey-West standard errors with maximum lag 2 months. We estimated an overall effect (and 95% confidence intervals) for all local outreach clinics by ethnic group, age group, and deprivation using random-effects meta-analysis. Between-study heterogeneity was reported using the I^2 statistic.
